# Precision-Engineered Construction of Proton-Conducting Metal–Organic Frameworks

**DOI:** 10.1007/s40820-024-01558-3

**Published:** 2024-12-11

**Authors:** Liyu Zhu, Hongbin Yang, Ting Xu, Feng Shen, Chuanling Si

**Affiliations:** 1 State Key Laboratory of Biobased Fiber Manufacturing Technology, Tianjin Key Laboratory of Pulp and Paper, Tianjin University of Science and Technology, 300457 Tianjin, People’s Republic of China; 2 State Key Laboratory of Pulp and Paper Engineering, South China University of Technology, 510640 Guangzhou, People’s Republic of China; 3 Agro-Environmenta Protection Institute, Ministry of Agriculture and Rural Affairs, 300191 Tianjin, People’s Republic of China; 4 Robustnique Co. Ltd., Block C, Phase II, Pioneer Park, Lanyuan Road, 300384 Tianjin, People’s Republic of China

**Keywords:** MOFs, Proton conduction, Porous materials, Fuel cells

## Abstract

Proton-conducting materials have attracted considerable interest because of their extensive application in energy storage and conversion devices. Among them, metal–organic frameworks (MOFs) present tremendous development potential and possibilities for constructing novel advanced proton conductors due to their special advantages in crystallinity, designability, and porosity. In particular, several special design strategies for the structure of MOFs have opened new doors for the advancement of MOF proton conductors, such as charged network construction, ligand functionalization, metal-center manipulation, defective engineering, guest molecule incorporation, and pore-space manipulation. With the implementation of these strategies, proton-conducting MOFs have developed significantly and profoundly within the last decade. Therefore, in this review, we critically discuss and analyze the fundamental principles, design strategies, and implementation methods targeted at improving the proton conductivity of MOFs through representative examples. Besides, the structural features, the proton conduction mechanism and the behavior of MOFs are discussed thoroughly and meticulously. Future endeavors are also proposed to address the challenges of proton-conducting MOFs in practical research. We sincerely expect that this review will bring guidance and inspiration for the design of proton-conducting MOFs and further motivate the research enthusiasm for novel proton-conducting materials.

## Highlights

- The effects of the size structure and stability of metal–organic frameworks (MOFs) on proton conduction are comprehensively summarized.
- Advanced strategies for constructing proton conduction MOFs are critically discussed.
- Challenges and opportunities for the development of novel proton-conducting MOFs are further outlined.

## Graphical abstract

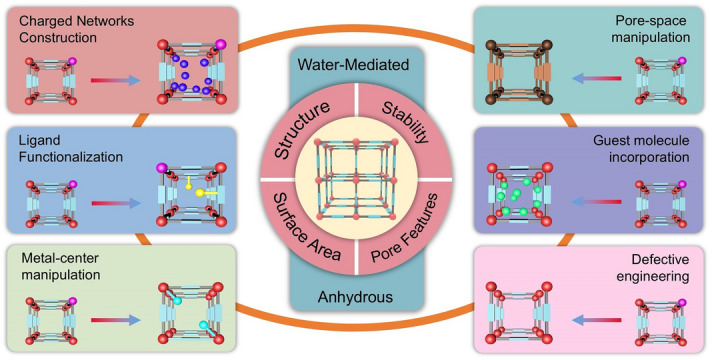

## Introduction

The rising global demand for energy coupled with environmental concerns stemming from fossil fuel usage has driven a global expansion of renewable and sustainable energy alternatives [1–6]. Renewable energy-based hydrogen energy, including production, storage, and conversion, is broadly recognized as a promising alternative for future energy sources [7–9]. In this process, proton exchange membrane fuel cells (PEMFCs) can convert the chemical energy in hydrogen into electrical energy efficiently in a carbon emission-free process, have aroused considerable attention due to their environmentally friendly and high-efficiency properties [10–12]. Notably, the polymer electrolyte membranes/proton exchange membranes (PEMs) as the core of PEMFCs, are the key to realizing their high performance, safety, and durability [13, 14]. To advance the FC iteration, several materials, such as Nafion and its substitute polymers, porous organic/inorganic/carbon materials, and inorganic/polymer composites have been successively designed and explored for different application scenarios [15–18]. Proton-conducting materials are evolving rapidly in this context. Indeed, two general design targets emerged in the research of proton-conducting materials for FCs: (i) developing novel materials suitable for operation in humid conditions (< 100 °C); (ii) developing efficient anhydrous proton conductors with performance independent of humidity conditions (> 100 °C).

**Fig. 1:**
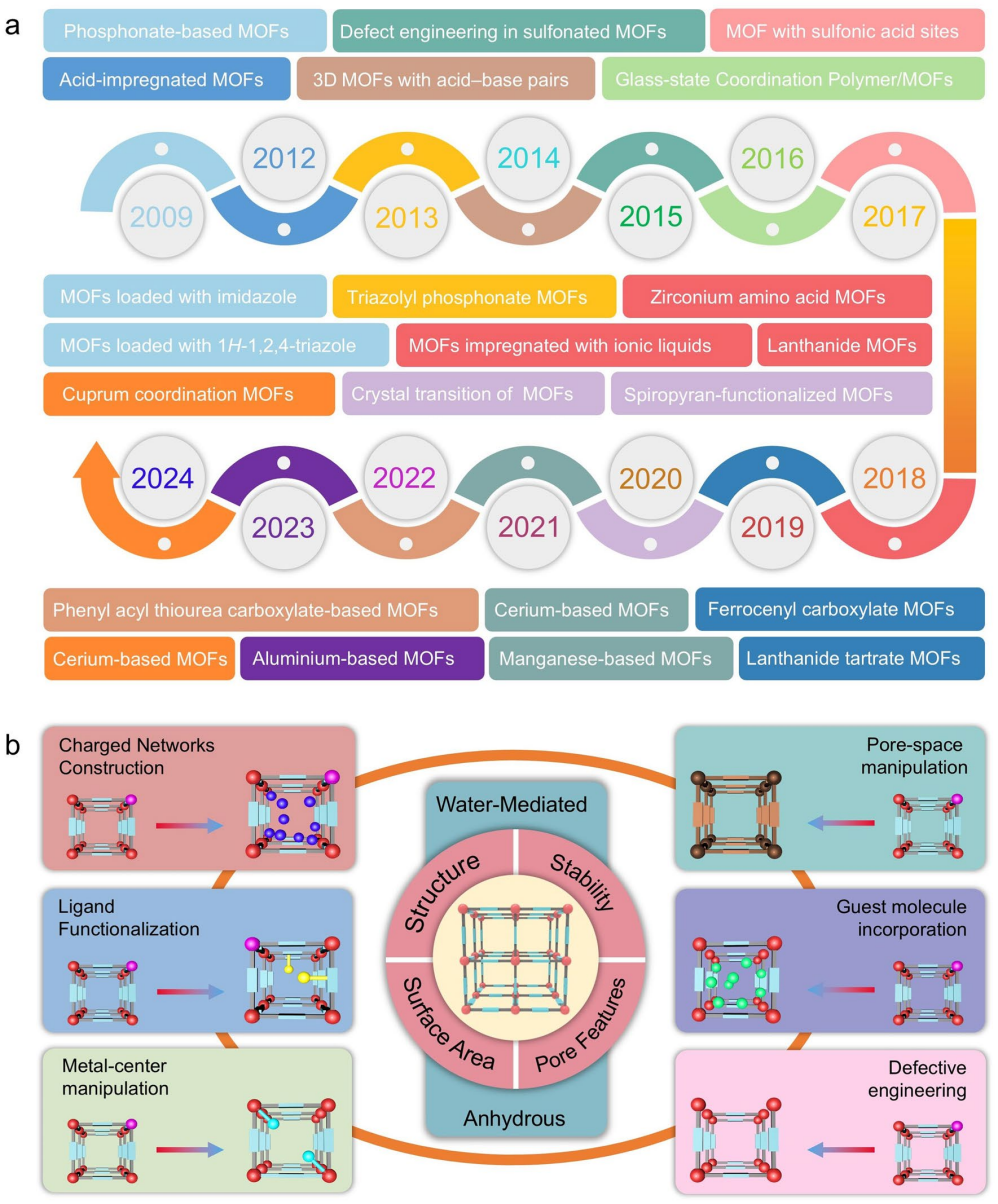
**a** Timeline of the development of representative proton-conducting MOFs (The color of the example module matches the color of the corresponding time module). **b** Design strategies for proton-conducting MOFs

During the iterative updating process of new energy technologies, porous materials capable of storing energy carriers or facilitating rapid ion conduction for efficient energy storage and conversion have been extensively investigated and analyzed [19–26]. Indeed, desirable morphology, suitable surface area, and exceptional functionality are decisive features for ion conduction and substrate reaction kinetics in this process [27–30]. Compared with conventional inorganic porous materials, MOFs feature tunable topologies/pore sizes, flexible customizability, permanent porosity, remarkable surface area, and organic–inorganic hybrid nature, which render them significantly superior in proton conduction [31–35]. Concurrently, the development of synthetic methods has also provided better control over the fabrication of MOFs with hierarchical microstructures [36, 37]. Particularly, MOFs with an extensive number of candidate structures (> 70,000) provide an unprecedented opportunity to further investigate the proton conduction mechanism and conduction behavior [38–44]. The proton-conducting MOFs were first presented in 1979 by Kanda et al., whereas the proton conduction mechanism of MOFs was unclear because of lacking crystallinity [45]. Until 2009, the proton conduction behavior in high-crystallinity MOFs was investigated by Kitagawa and coworkers [46]. During the past two decades, proton-conducting MOFs have been evolving rapidly with numerous novel structures and types (Fig. 1a). In general, protons diffuse mainly through a network of hydrogen bonds between the guest molecules and MOF framework [47–49]. Thus, the construction of proton-conducting MOFs focuses on the creation of a high-density hydrogen bonding network and an increase in the concentration of mobile protons [50, 51].

Based on previously reported strategies for constructing proton-conducting MOFs, proton conduction MOFs are categorized into six types according to the hopping site and proton source (Fig. 1b): (i) formation of anion (SO_4_^2−^), ammonium (NH_4_^+^, Me_2_NH_2_^+^), or hydronium (H_3_O^+^) counterionic inclusions leads to the creation of charged framework during the synthesis of MOFs [52]; (ii) insertion of functional molecules (*e.g.*, imidazole, EtOH, and H_2_O) by coordination functionalization of metal centers of MOFs [53]; (iii) incorporation of uncoordinated functional groups (*e.g.*, –SO_3_H, –PO_3_H_2_, –COOH, –NH_2_) in organic ligands into functional molecules [1]; (iv) incorporation of guest molecules such as metal–organic polyhedra (MOP) molecules, polyoxometalate (POM), protic organic molecules, and acid molecules in MOFs [54]; (v) construction of defective structures in MOFs to create additional conduction pathways [55]; (vi) impartation of hydrophilicity to MOFs enhances humidity-induced proton conductance sensitivity [56]; indeed, the reported proton-conducting MOFs are more complicated because the various combinatorial components act simultaneously to produce synergistic effects. In addition, in terms of the type of proton conduction, research on proton conduction MOFs can be categorized into two types: (i) water-mediated proton conduction and (ii) anhydrous proton conduction. The proton conduction mechanism has been investigated extensively using H_2_O as a proton conduction medium. Moreover, two proton conduction mechanisms have been proposed considering the diffusion motion of protons: the Grotthuss mechanism and the vehicle mechanism (detailed description in Sect. 3).

Owing to the inherent advanced features and properties, proton-conducting MOFs have made extensive and considerable progress, and an impressive number of novel MOFs with excellent conductivity are rapidly emerging. Motivated by such research passion, proton-conducting MOFs became a landmark in the application of MOFs. In this review, the effect of the dimensional structure and stability of MOFs on proton conduction is first discussed in detail from the basic properties of MOFs. Starting from the conduction mechanism and behavior, the advanced strategies for constructing proton-conducting MOFs are discussed in detail and critically with representative examples. Additionally, based on the insightful consideration and understanding of proton-conducting MOFs, the opportunities and challenges for the further construction of novel proton-conducting MOFs are summarized and outlined. We sincerely hope that this review will offer several accessible inspirations for the sophisticated design and potential applications of proton conduction MOFs.

## Dimensional Structure and Characteristics of MOFs

**Fig. 2:**
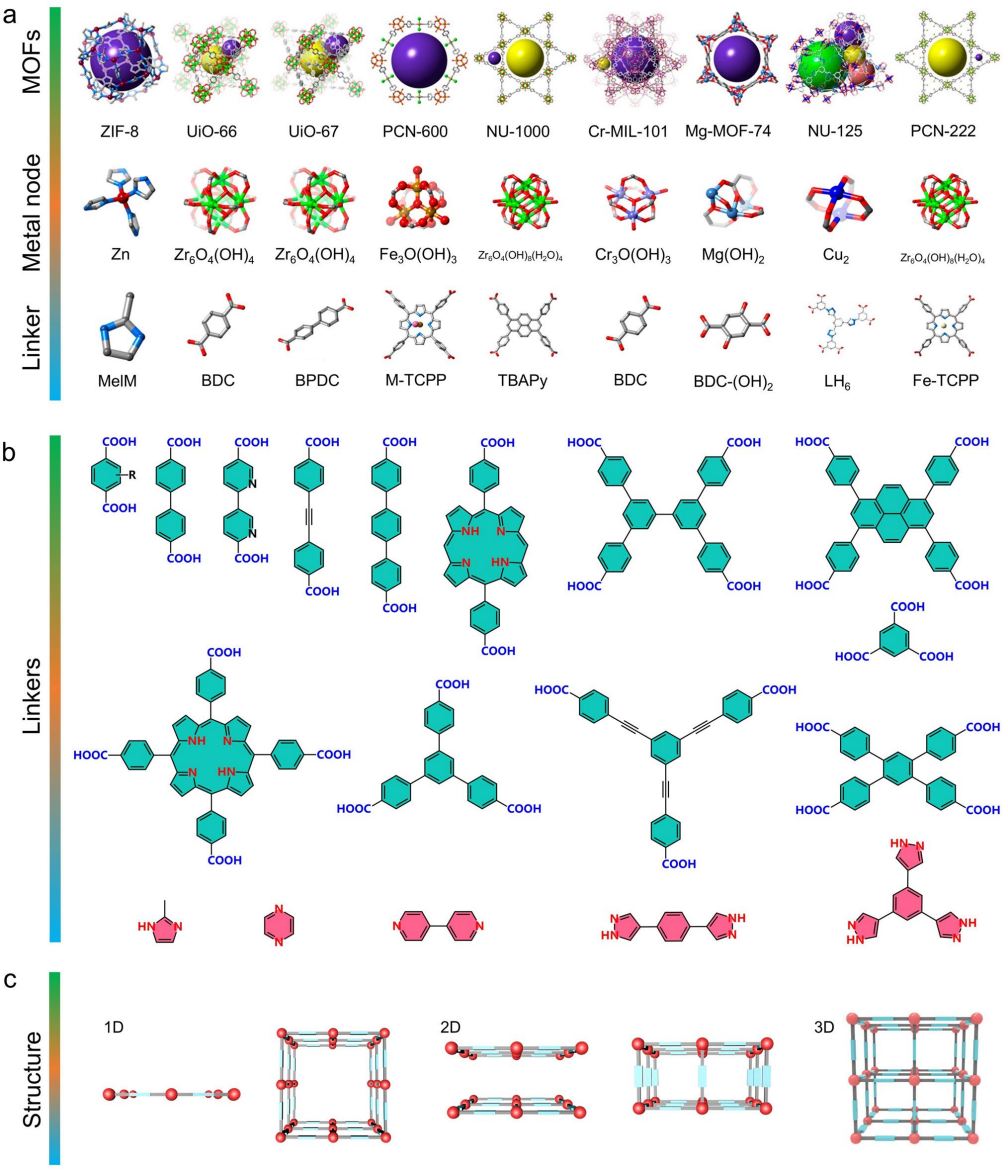
**a** Schematic diagram of representative MOF structures, corresponding nodes, and links. **b** Schematic diagram of representative MOF linkers. **c** Spatial structure of MOFs (1D, 2D, and 3D)

The last decade showed explosive growth in the production, characterization, and research of MOF materials. In general, MOFs are constructed through the attachment of metal-containing units [secondary building units (SBUs)] with organic linkers (nitrogen/carboxylic acid-containing ligands) by utilizing strong bonds to form crystal frameworks with permanent porosity [33, 57–59]. The diversity of SBUs and organic linkers provides a deep structural foundation for the crystalline structure and multifunctionality of MOFs (Fig. 2). Briefly, MOFs can be adapted to have customized structure and function for a specific application by flexible selection of SBUs and connectors since the structure of MOFs is determined by the geometry of SBUs and the sizes/shapes of the organic ligands [42, 60, 61]. Moreover, three items are particularly important in driving the iterative updating of MOF chemistry during the development of MOF materials: (i) the geometrical rationale of construction is achieved by connecting SBUs to rigid shapes such as octahedra or squares, instead of the simple spacer and node construction of early ligand networks where individual atoms are connected by coordination linkers; (ii) post-synthesis modification of MOFs containing metal–organic complexes and organic units by reaction with linkers can effectively change the pore environment; (iii) the incorporation of multiple organic functionalities in a single framework of MOFs provides numerous opportunities to engineer complexity into the MOF pores. Meanwhile, MOFs possess different structural dimensions such as 1D, 2D, and 3D frameworks [62–64]. That is, the controlled synthesis and structural characterization of MOFs provide a deep foundation for their application in proton conduction. In this section, the different dimensional structures of MOFs and their proton conductivity are highlighted, and examples are given to illustrate their representativeness.

### Structure

#### 1D Structure

**Fig. 3:**
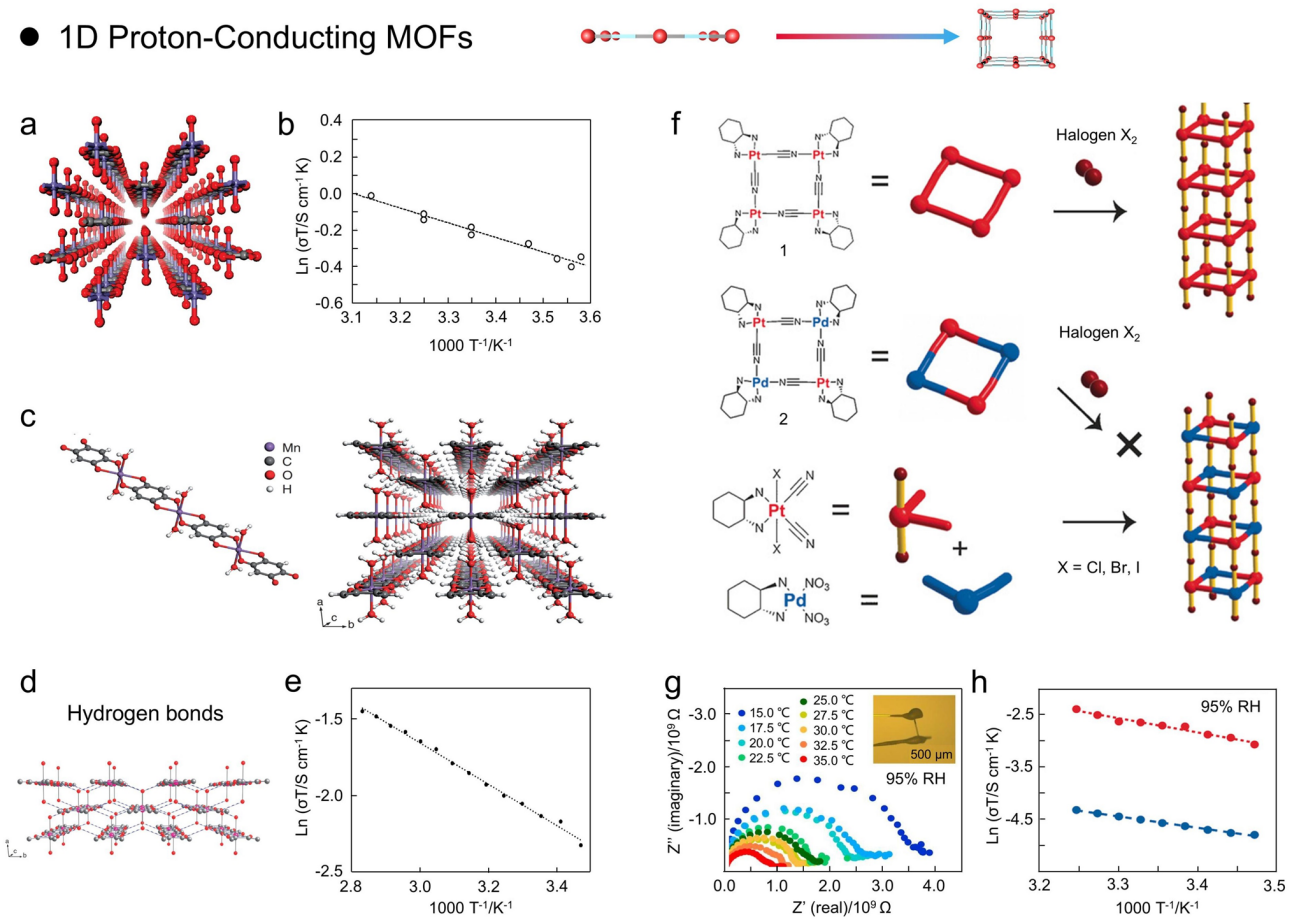
**a, b** Crystalline structure and temperature-dependent Arrhenius-type plot of 1D ferrous oxalate dehydrate. (Reproduced with permission from Ref. [46]. Copyright 2009, American Chemical Society) **c** Crystal structure of 1D Mn(dhbq)·(H_2_O)_2_. **d** Hydrogen bonds lie perpendicular to the 1D chain in 1·2H_2_O. **e** Temperature-dependent Arrhenius-type plot of 1D Mn(dhbq)·(H_2_O)_2_. (Reproduced with permission from Ref. [66]. Copyright 2009, Chemical Society of Japan) **f** Schematic diagram of the facile synthesis of heterometallic square 1D nanowires. **g** Nyquist plots of [Pt(dath)(CN)Br]_4_(NO_3_)_4_ at elevated temperature and 95% RH. **h** Temperature-dependent Arrhenius plots of proton conductivities of [Pt(dath)(CN)Br]_4_(NO_3_)_4_ and [Pd(dath)][PtX_2_(dath)(CN)_2_]_2_(NO_3_)_4_ at 95% relative humidity. (Reproduced from Ref.[69]. Copyright 2016, Wiley–VCH)

The reduced size and orientated framework structure of 1D nanomaterials contribute to the unique characteristics that differ from other dimensional nanomaterials. In this regard, Yamada and coworkers reported 1D ferro-oxalate MOFs [Fe(ox)·2H_2_O] with superior proton conductivity (Fig. 3a) [46]. Briefly, Fe(ox)·2H_2_O prepared from a simple mixture of oxalic acid and ferrous sulfate has a 1D structure where Fe^2+^ is linked by oxalic acid ligand and two water molecules are liganded to Fe^2+^. Notably, the ligand water molecules are stronger in acidity and more readily available for protons than the free water molecules. That is, the mobile protons in ordered water arrays induced diffusion of protons through hydrogen bonding networks more readily. Therefore, Fe(ox)·2H_2_O has a proton conductivity of 1.3 × 10^–3^ S cm^−1^, a comparatively high value without any exogenous strong acid species at room temperature. Indeed, this exceptional proton conduction property is ascribed to the orderly coordination of water molecules inside the Fe(ox)·2H_2_O and the long-range ordered nanoscale interfaces. Moreover, the activation energy (*E*_*a*_) of Fe(ox)·2H_2_O was 0.37 eV, which was consistent with the concept of a superionic conductor (Fig. 3b). At a deeper level, the strong interactions between the skeletons and guest molecules are responsible for the enhanced transport of protons on the nanoscale.

In general, ionic conduction is significantly strengthened when the conduction path is confined to the nanoscale [65]. Therefore, from this direction, Kitagawa and coworkers presented a 1D Mn-based MOF [Mn(dhbq)(H_2_O)] with excellent proton conductivity (Fig. 3c) [66]. Briefly, Mn(dhbq)(H_2_O)_2_ was fabricated at room temperature through slow diffusion of an aqueous solution comprising H_2_dhbq and manganese sulfate. Interestingly, 1D Mn(dhbq)(H_2_O)_2_ with alternating arrangements of divalent manganese ions and dhbq can adsorb two equal amounts of water molecules under humidified conditions. Remarkably, the proton conductivity of Mn(dhbq)(H_2_O)_2_ is lower than that of Fe(ox)·2H_2_O by an order of magnitude, even though they have comparable crystal structures and stacked hydrogen bonding networks (Fig. 3d). Moreover, the activation energy (*E*_*a*_) of Mn(dhbq)(H_2_O)_2_ estimated from proton conductivity was 0.26 eV, lower than Fe(ox)·2H_2_O (0.37 eV) (Fig. 3e). This phenomenon demonstrates that proton conduction is significantly influenced by the Lewis acidity of the metal centers of MOFs, the distance between the coordinated water molecules and the ligands.

Achieving high mobility of ions in crystalline solids is challenging since ions have a tendency to be tightly anchored in crystals through strong interactions [67]. To address this critical issue, Horike and coworkers synthesized a 1D ionic crystal [Zn(HPO_4_)(H_2_PO_4_)_2_](ImH_2_)_2_ with superior anhydrous proton conductivity consisting of protonated imidazole and Zn^2+^ phosphate [68]. Plastic crystal refers to a compound material with a long-range ordered crystal structure, such as some hydrogen and lithium-ion conductors. The coordination network of [Zn(HPO_4_)(H_2_PO_4_)_2_](ImH_2_)_2_ was composed of two orthophosphates (H_2_PO_4_^−^ and HPO_4_^2−^) and tetrahedrally coordinated Zn^2+^ and formed an extended 1D network. Briefly, the 1D network containing the [Zn(HPO_4_)(H_2_PO_4_)_2_] unit has a charge of -2. To maintain charge equilibrium, the impregnated imidazole molecules were protonated and formed an ionic crystal system with multiple hydrogen bonds in the spaces between the 1D crystal chains. Meanwhile, the proton conductivity tests were performed within the temperature range 25–140–40 °C and revealed that a nonlinear increase in conductivity triggered by the movement of mobile ions was observed at 55 °C. This phenomenon was mainly attributed to the increased mobility in ImH_2_^+^. Moreover, this 1D ionic plastic crystal also showed the proton conductivity of 2.6 × 10^–4^ S cm^−1^ at 130 °C and had an *E*_*a*_ of 0.47 eV in the temperature range of 40–130 °C (Grotthuss mechanism). This result presented a strategy for constructing ionic plastic crystals via ligand networks and counter cations/anions, which would facilitate the development of the field of plastic crystals and solid ionic conducting materials.

1D nanowires based on coordination bonds have recently aroused exceptional attention because of their readily modifiable structures and their physicochemical properties can be systematically controlled by substituting structural components such as organic ligands and metal ions [64]. In this regard, Otsubo and coworkers investigated the proton conduction properties of metal–organic nanowires built from (platinum, Pt) homometallic and (palladium/platinum, Pt/Pd) heterometallic systems by ligand-driven self-assembly (Fig. 3f) [69]. Briefly, a homometallic nanowire (Pt) was constructed by attaching Pt monomers to create square rings and then connecting the rings with halogen atoms by oxidative polymerization to create metal halide bridges along the *b* direction, while the heterometallic nanowire (Pt/Pd) was prepared by self-assembly of halogen-linked Pd and Pt monomers. Notably, these two 1D nanowires display a network of hydrogen bonds constructed synergistically by NH_2_, NO_3_^−^, and H_2_O, thereby generating the expected proton conductivity. Therefore, the homometallic nanowire (Pt) and heterometallic nanowire (Pt/Pd) exhibited the proton conductivities of 1.29 × 10^–5^ S cm^−1^ (*E*_*a*_ = 0.54 eV) and 1.54 × 10^–7^ S cm^−1^ (*E*_*a*_ = 0.43 eV) at 85 °C and 95% RH, respectively (Fig. 3g, h). Although these two nanowires have similar spatial structures, their conductivities differ by two orders of magnitude due to the different electronic states and charge modulation of the Pt and Pd atoms inside the nanowires, which also clearly demonstrates the role of the electronic states in controlling the proton conductivity in ligand-driven 1D nanocrystal structures.

#### 2D Structure

**Fig. 4:**
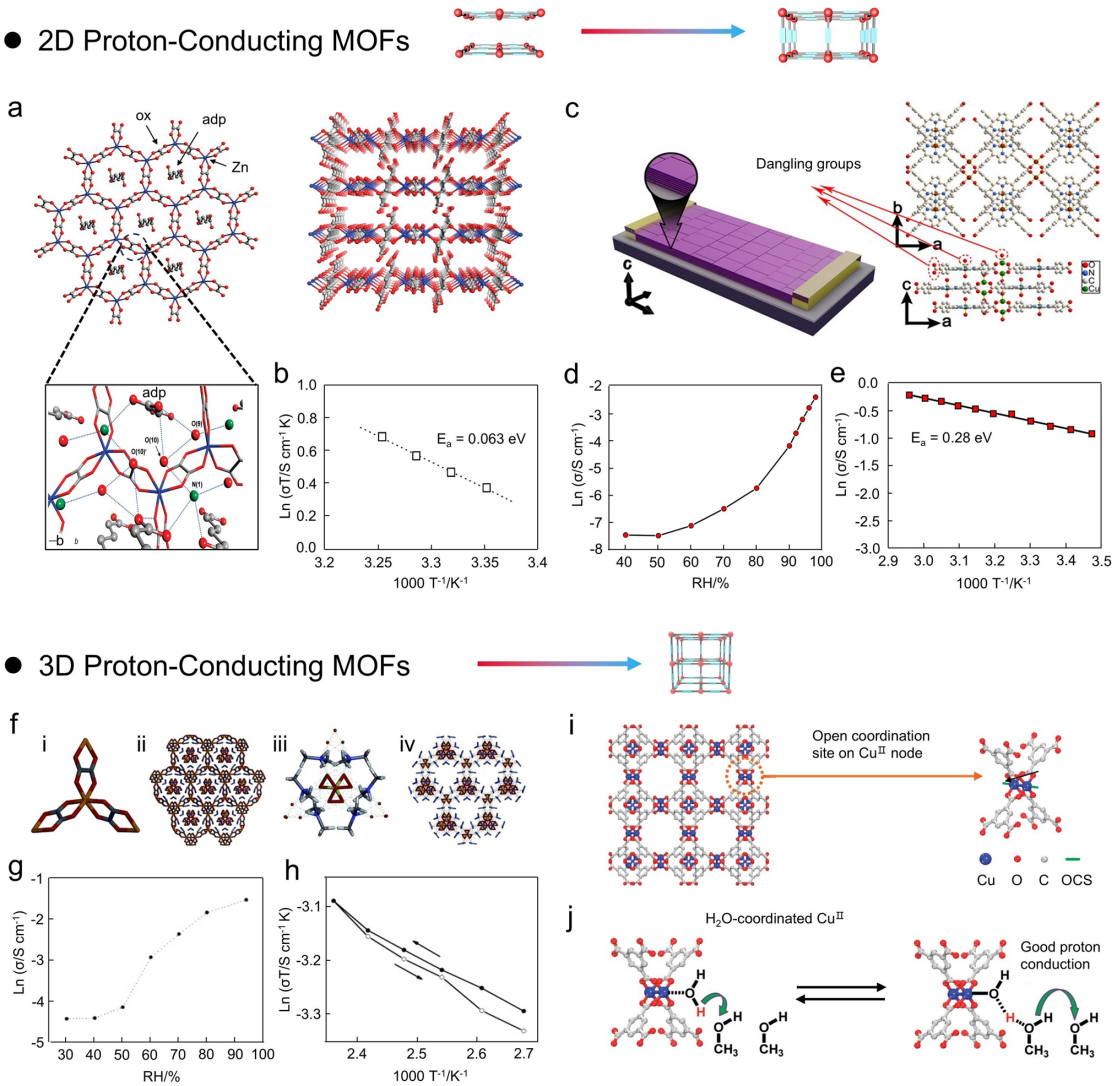
**a** Schematic diagram of the honeycomb structure and perspective view of (NH_4_)_2_(adp)[Zn_2_(ox)_3_]·3H_2_O. **b** Arrhenius plots of (NH_4_)_2_(adp)[Zn_2_(ox)_3_]·3H_2_O. (Reproduced with permission from Ref. [73]. Copyright 2009, American Chemical Society) **c** Schematic of the 2D MOF membrane for electrical measurements and crystal structure of the MOF membrane. **d, e** Proton conductivities and Arrhenius plots of 2D MOF membrane under different RH conditions. (Reproduced with permission from Ref. [77]. Copyright 2013, American Chemical Society) **f** Structure of tris-chelated *D*_3_-symmetric [Zn_2_(ox)_3_]^2−^ subunit (i), the crystal structure of ([(Me_2_NH_2_)_3_(SO_4_)_2_][Zn_2_(ox)_3_])_n_ (ii), the hydrogen bonding interactions between sulfate anions and dimethyl ammonium cations (iii), the structure of 3D [(Me_2_NH_2_)_3_(SO_4_)]^+^_n_ net (iv). **g, h** Arrhenius plot and time-dependent proton conductivity of ([(Me_2_NH_2_)_3_(SO_4_)_2_][Zn_2_(ox)_3_])_n_. (Reproduced with permission from Ref. [80]. Copyright 2014, Wiley–VCH) **i** Schematic diagram of the HKUST-1 structure along the (100) direction and nodes. **j** Schematic illustration of proton transfer from Cu^II^ centers coordinated to water. (Reproduced with permission from Ref. [88]. Copyright 2012, American Chemical Society)

In terms of dimensional properties, 2D layered structures typically have greater degrees of freedom than 3D structures due to their weak interlayer bonding, and their controlled interlayer stacking and sliding motion provide enormous opportunities to modulate their physicochemical properties, especially for 2D coordination compounds pre-designed according to the coordination geometry of metal species and ligand shapes can generate pores by stacking of π-π interactions between the ligands [70–72]. The efficient proton conduction is then induced by the conducting medium in the pores and layers of the 2D layered materials. In 2009, Kitagawa and coworkers constructed a 2D Zn-based MOFs (NH_4_)_2_(adp)[Zn_2_(ox)_3_]·3H_2_O with high proton conductivity by introducing water molecules as a conducting medium in the framework of oxalate-mediated anionic layers (Fig. 4a) [73]. Notably, the formed [Zn_2_(ox)_3_]^2−^ features a 2D laminar structure similar to a honeycomb, with guest water molecules, NH_4_^+^, and adipic acids positioned in the interlayer space. Through these abundant hydrogen bonding networks, (NH_4_)_2_(adp)[Zn_2_(ox)_3_]·3H_2_O can achieve the proton conductivities of 8 × 10^–3^ and 6 × 10^–6^ S cm^−1^ at 98% RH and 70% RH, respectively. The *E*_*a*_ of (NH_4_)_2_(adp)[Zn_2_(ox)_3_]·3H_2_O was 0.63 eV, which represented a typical Grotthuss proton conduction mechanism (Fig. 4b). Indeed, the efficient proton conduction of (NH_4_)_2_(adp)[Zn_2_(ox)_3_]·3H_2_O is attributed to higher carrier density of water molecules, adipic acid, and counterions.

Some rare MOF minerals can exhibit superior proton conductivity and high thermodynamic stability comparable to known oxalate MOFs. Navrotsky and coworkers reported a rare oxalate MOF mineral material with 2D zhemchuzhnikovite (ZH) and stepanovite (ST) with excellent proton conduction properties [74]. It is worth noting that the ST has two polycrystalline forms (ST1 and ST2) determined by the synthesis conditions, in which slow evaporation produces hexagonal crystals (ST1) and fast evaporation produces elongated crystals resembling (ST2). In terms of proton conductivity, ZH exhibits a conductivity of 3 × 10^–3^ S cm^−1^ at 25 °C and 90% RH, which is one order of magnitude higher than ST1. Moreover, it is noteworthy that the ST2 is unable to perform impedance testing at high RH conditions (> 70% RH) on account of deliquescence. Under low RH conditions, the *E*_*a*_ of ST2 (1.31 eV) is significantly higher than ST1 (0.59 eV) and ZH (0.37 eV). Indeed, the variation in proton conductivity between ST1, ST2, and ZH may be associated with the different topologies of the hydrogen bonding frameworks created by the water guests and Mg(H_2_O)_6_^2+^.

Generally, proton-conducting solids can be classified into two categories: intrinsic conductors and hybridized conductors. Indeed, the critical factors for obtaining intrinsic proton conductivity are structure, acidity, and carrier concentration [75]. To obtain MOFs with intrinsic proton conduction properties, Horike and coworkers fabricated a 2D coordination polymer [Zn(H_2_PO_4_)_2_(TzH)_2_]_n_ consisting of Zn^2+^, orthophosphates (H_2_PO_4_), and 1,2,4-triazole (TzH, C_2_H_3_N_3_), and proved the intrinsic proton conductivity in the coordination network [76]. The extended 2D sheets parallel to the *ab* plane are formed by octahedral coordination of Zn via two mono-coordinated orthophosphates and four bridged TzHs. The layers are connected by the internal/external hydrogen bonds along the *c*-direction of mono-coordinated orthophosphate stacks. That is, ligands in the layer are connected via extended hydrogen bonds, allowing [Zn(H_2_PO_4_)_2_(TzH)_2_]_n_ to exhibit an intrinsic proton conductivity of higher than 10^–4^ S cm^−1^ at 150 °C without other guests. Notably, the spacing of protons in the interlayers of 2D structure is crucial for intrinsic proton conduction. Further, Kitagawa and coworkers synthesized a highly oriented crystalline MOF nanofilm (Cu-TPP) with ultra-high proton conductivity and low activation energy (Fig. 4c) [77]. Briefly, MOF nanosheets (Cu-TPP) with high aspect ratios (400 nm in diameter and 15 nm thickness) were prepared by H_2_TCPP and Cu(NO_3_)_2_ as building blocks, and Cu-TPP membrane was further formed by deposition of the nanosheets onto Cr/Au electrode. Notably, the proton conductivity of the Cu-TPP membrane was 3.9 × 10^–3^ S cm^−1^ at 25 °C and 98% RH, and *E*_*a*_ was 0.28 eV (Fig. 4d, e). Indeed, this high proton conductivity and low *E*_*a*_ have benefited from the functional groups in the 2D layers of Cu-TPP, such as the liganded water molecules and the terminal carboxyl groups of ligands. That is, the surface and size of MOF crystals are of significant importance for proton conduction, especially when the size of MOF crystals is reduced to the nanoscale, where the surface properties of nanocrystals could take a predominant role in proton conduction.

#### 3D Structure

3D MOFs typically exhibit unexpected functions and characteristics due to their special spatial properties [78, 79]. Ghosh and coworkers developed a 3D MOF ([(Me_2_NH_2_)_3_(SO_4_)_2_][Zn_2_(ox)_3_]_n_) that can achieve efficient proton conduction under anhydrous and humid conditions (Fig. 4f) [80]. This 3D MOFs were composed of the anionic framework [Zn_2_(ox)_3_]^2−^_n_ interpenetrated with the cationic supramolecular network [(Me_2_NH_2_)_3_(SO_4_)]^+^_n_. Therefore, the pure phase of 3D MOFs showed high proton conductivities of 1.0 × 10^–4^ S cm^−1^ at 150 °C and 4.2 × 10^–2^ S cm^−1^ at 25 °C and 98% RH (Fig. 4g). Meanwhile, similar *E*_*a*_ values can be obtained at heating (0.129 eV) and cooling (0.130 eV), respectively (Fig. 4h). Indeed, this excellent proton conductivity originates from the transfer of protons along hydrogen-bonded sulfate anions and dimethylammonium cations, high/uniform carrier loadings, as well as highly symmetric and ordered arrangement of proton carriers. More importantly, such 3D MOFs constructed from cheap materials have inherent water-assisted and anhydrous high proton conductivity, making them ideal for building proton sensors and solid-state electrolytes.

Generally, two methods can be employed to generate proton conductivity in MOFs: (i) incorporating functional groups such as phosphonic acid, hydrochloric acid, sulfonic acid, or carboxylic acid into the framework linker as channel-accessible sites [81–83]; (ii) introducing proton donors/carriers such as 1H-1,2,4-triazole, carboxylic acid, or ammonium ion species into the nanopores of formed MOFs [84–87]. In this regard, Farha and coworkers proposed a strategy employing coordination chemistry to endow 3D MOFs (HKUST-1) with excellent proton conductivity properties (Fig. 4i) [88]. 3D HKUST-1 contains 1,3,5-benzenetricarboxylate (BTC) struts and Cu^II^-paddlewheel type nodes with accessible Cu^II^ sites to coordinate solvents or other molecules. Notably, each Cu ion on the HKUST-1 node contains an open ligand site that is dominated by solvent molecules and can be exchanged with various solvent molecules, such as H_2_O, ethanol, methanol, and CH_3_CN by post-synthetic modifications. Despite the neutrality of MOFs, Cu^II^ can enhance the proton conductivity by rendering the ligand water molecules sufficiently acidic to provide protons to the methanol molecules that fill the pores. Especially, the proton conductivity of HKUST-1 was improved about 75-fold when the liganded acetonitrile molecules were substituted by water molecules (15 μS cm^−1^). The enhancement of proton conductivity was ascribed to increased proton donation of methanol by the liganded water, as the *pK*_*a*_ value decreased after the water molecule was liganded to the metal center (Fig. 4j). Although this research is limited to several HKUST-1 variants, this novel approach presents an important theoretical basis for enhancing proton conductivity in MOFs.

### Stability

Although numerous new structures of MOFs have been presented in the past two decades, the stability enhancement of MOFs has been a caustic problem, such as water/chemical/thermal/mechanical stability [89–92]. Briefly, water and polar solvents disrupt the MOF structure through the solvation of metal ions, while hydrogen and hydroxide ions are prone to attack the metal–ligand bonds of MOFs [93, 94]. Moreover, the strong nucleophilic reagents in water also tend to displace the ligand and break down the structure of MOFs depending on their strong coordination tendency. In this regard, considerable efforts have been made to address this challenge in recent years.

#### Water and Chemical Stability

**Fig. 5:**
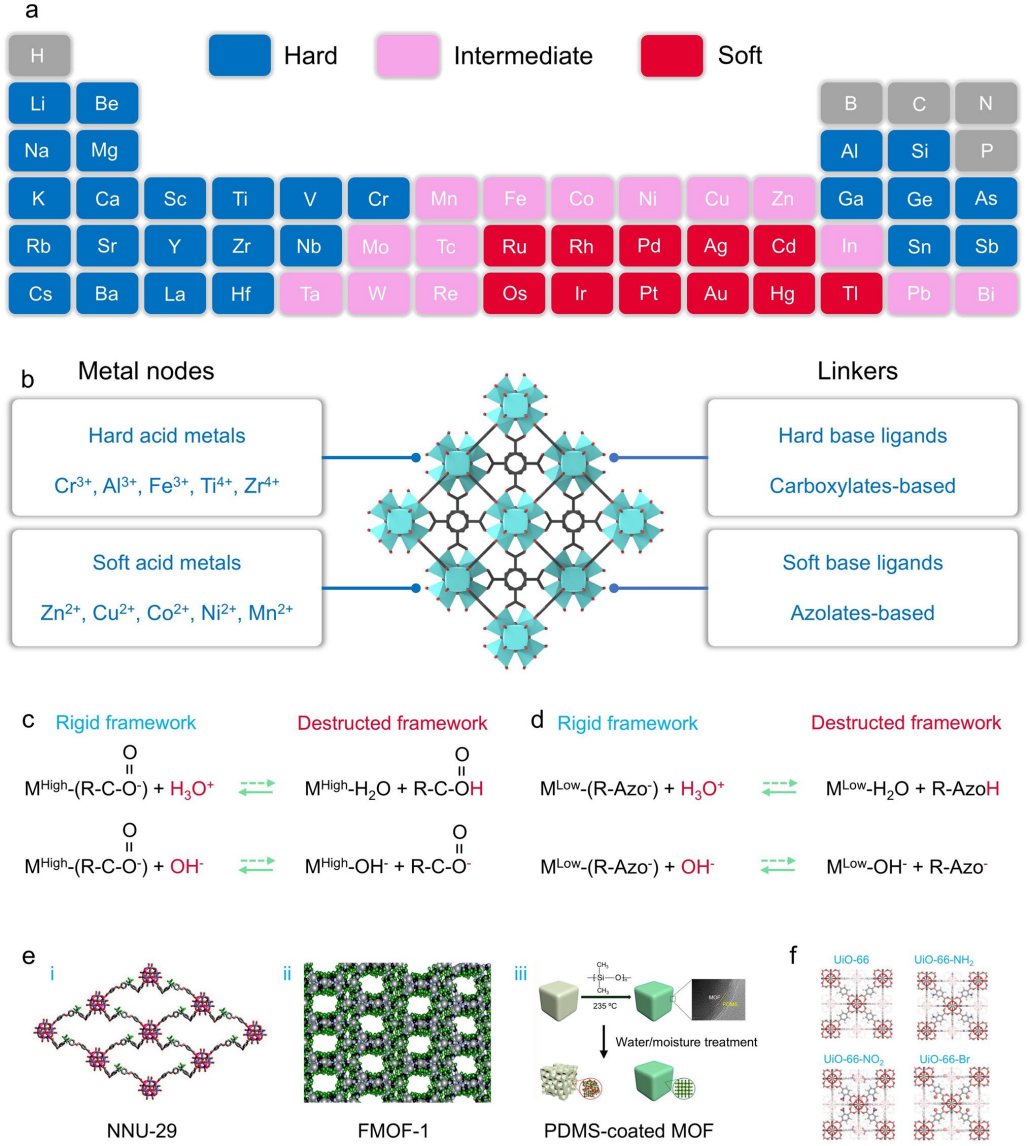
**a** Divide metals in the periodic table into hard acids, intermediate acids, and soft acids. **b** Framework structure of hard acid metal-based MOFs represented by UiO-66 and common hard/soft acids/ligands. **c, d** Metal–ligand substitution reaction pathways between protons/hydroxides and MOFs (Reproduced with permission from Ref. [94]. Copyright 2021, Cell Press) **e** Examples of MOFs with high chemical stability (fluorinated MOF and PDMS-coated MOF). (Reproduced with permission from Refs. [100, 102, 103] Copyright 2019, American Chemistry Society; Copyright 2013, American Chemistry Society; Copyright 2014, American Chemistry Society) **f** Structural of UiO-66, UiO-66-NH_2_, UiO-66-NO_2_, and UiO-66-Br. (Reproduced with permission from Ref. [106]. Copyright 2010, American Chemistry Society)

Indeed, the properties of metal–ligand bonds dominate the stability of MOFs [95, 96]. In general, metal cations are categorized into hard, intermediate, and soft acids depending on *pK*_*b*_ values, and basic ligands without protons are categorized into hard, medium-strength base, and soft bases according to their *pK*_*a*_ values (Fig. 5a). Generally, MOFs with inter-matched metal–organic linkers typically have higher stability (Fig. 5b). In this regard, carboxylate ligands are the common hard bases and are typically combined with highly valent hard acid metal ions such as Cr^3+^, Al^3+^, Fe^3+^, Ti^4+^, and Zr^4+^ to synthesize stable MOFs. It is noteworthy that metal cations with higher charge densities usually create more polarized ligand bonds under the same coordination environment, which facilitates the formation of more stable MOFs [95, 97]. The MIL series of MOFs, which combine high-valent metal cations and carboxyl ligands represents a class with high stability [98]. For example, the metal–ligand bond is strengthened by the strong coulombic interactions and polarization between the negatively charged carboxylic acid linker and the highly oxidophilic Zr^4+^ in UiO-66.

MOFs with weak metal–ligand bonds are susceptible to decomposition in aqueous environments due to ligand substitution or hydrolysis. Briefly, water molecules could be intercalated between the ligand and metal during the ligand substitution (Eq. 1), while the ligand and metal can be replaced by hydroxides and hydrogen ions during hydrolysis (Eq. 2), respectively [98, 99]. In general, MOFs containing hard base ligands and hard acidic metals usually show lower stability under alkaline conditions and higher stability under acidic conditions, while MOFs containing soft base ligands and soft acidic metals show the opposite trend (Fig. 5c, d). In particular, hydrogen ions tenderly compete with metal ions for organic ligands under acidic conditions. These ligands are deprotonated even at lower pH values due to the relatively low *pK*_*a*_ values of hard base ligands. Nevertheless, higher *pK*_*a*_ values of soft base ligands tend to maintain a protonated state at low pH. Therefore, protons are prone to displace metal ions more readily resulting in the decomposition of the structures of MOFs under such microscopic reaction pathways. In addition, the presence of hydroxides can complete the ligation of the metal ion with the organic ligand, where the hard acid metal binds tightly to the hydroxide, while the soft acid metal remains coordinated with the ligand.

1 $$\left(M-L\right)+{\text{H}}_{2}\text{O}\to M-O{\text{H}}_{2}\cdots L$$

2 $$\left(M-L\right)+{\text{H}}_{2}\text{O}\to M-\left(\text{OH}\right)+(L-H)$$

From another perspective, the hydrophilicity of MOFs also has a particularly significant impact on their stability. In this regard, endowing MOFs with excellent hydrophobicity through a post-modification strategy (such as ligand functionalization and surface coating) is a classical and effective approach to enhancing their stability [100, 101]. For example, Lan and coworkers combined polyoxometalate (POM) with hydrophobic ligands (trifluoromethyl, CF_3_) to synthesize a POM-based MOFs (NNU-29) with superior resistance to acids and bases (Fig. 5e) [102]. The hydrophobic CF_3_ groups surrounding the hydrophilic POM nodes give the synthesized NNU-29 good hydrophobicity and prevent the erosion of water molecules. Similarly, Chabal and coworkers replaced the hydrogen atoms in 1,4-benzenedicarboxylate (BDC) ligand with fluorine atoms, thus equipping the synthesized MOFs (FMOF-1) with fully fluorinated nanopores (Fig. 5e) [103]. The hydrophobicity induced by the perfluorinated nanopores gives FMOF-1 excellent stability. Moreover, Cohen and coworkers utilized long alkyl substituents for post-synthetic covalent modification of MOFs enabling these hydrophilic MOFs to be easily converted into hydrophobic or superhydrophobic materials, thus exhibiting superb resistance to aqueous environments [104]. Li and coworkers introduced methyl groups into the carboxylate ligands allowing the synthesized Zr-based MOFs (BUT-12 and BUT-13) to exhibit higher stability in the concentrated HCl/NaOH or boiling water [105]. Briefly, the presence of hydrophobic methyl groups can prevent water molecules from intruding into the structure of MOFs and increase the electron density of the carboxyl oxygen atoms by providing electrons to the benzene ring in the ligand, resulting in more polarized and stronger Zr-O bonds. It is worth noting that increasing the hydrophobicity of MOFs through ligand functionalization may have a negative impact on their porosity. Therefore, growing hydrophobic coatings on the MOF surface can strengthen their stability without affecting porosity. In this regard, Jiang and coworkers modified hydrophobic polydimethylsiloxanes (PDMS) on the MOF surface (ZnBT, HKUST-1, and MOF-5) by a facile gas-phase deposition technique to significantly enhance their water resistance (Fig. 5e) [100]. More importantly, these fragile MOFs retained their original crystalline properties and pore characteristics well after being coated by PDMS.

#### Thermal and Mechanical Stability

The thermal stability of MOFs also deserves special attention to cope with applications with high-temperature requirements. In most cases, the thermal degradation of MOFs is the consequence of metal node-organic linker bond breakage and subsequent linker combustion generation. Therefore, the thermal stability of MOFs is highly determined by the connectivity and strength of the metal–ligand bonds. For example, the crystal collapse temperature (*T*_*decomp*_) of UiO-66 is about 540 °C [106]. In contrast, the *T*_*decomp*_ of the UiO-66-NH_2_ and UiO-66-NO_2_ is about 350 °C, and the largest spatial site resistance of UiO-66-Br and the smallest site resistance of UiO-66 had comparable *T*_*decomp*_ (Fig. 5f). That is, the *T*_*decomp*_ of UiO-66 is not dependent on either steric repulsion effects or electronic effects. In this process, MOFs with highly symmetric structures and higher crystal densities may have higher thermal stability. Moreover, the thermal stability can be improved by enhancing the strength of metal–ligand bonds using equivalent oxyanion-terminated linkers with higher valent metal centers, such as Ti(IV), Zr(IV), Al(III), and Ln(III) [107, 108]. Another way to enhance thermal stability is to change the composition of the linker pendant groups [109].

From an engineering point of view, the mechanical stability of MOFs is another significant aspect of MOFs under pressure or vacuum in industrial and practical applications [92, 110]. For example, the structural instability of MOFs under vacuum conditions may cause phase transition or partial collapse of nanopores. In this regard, solvent evacuation and solvent exchange are commonly employed to activate MOFs and avoid structural collapse. Briefly, the substitution of solvents with lower surface tension for solvents with higher surface tension, as well as the removal of solvents, can facilitate the enhancement of the mechanical stability of MOFs. Specifically, Zr-based MOFs usually exhibit excellent mechanical stability due to the high strength of node-linker bonds and the high coordination number of the nodes. Indeed, the geometry of MOFs is also related to mechanical stability. In this regard, some theoretical computational studies have shown that shorter linkers contribute to enhanced mechanical stability [96, 111–113].

## Proton Conduction Mechanism

In general, two mechanisms have been proposed to explain the proton conduction process: Grotthuss mechanism and vehicle mechanism [49, 114–116]. The Grotthuss mechanism explains the proton conductive behavior in the hydrogen bonding network of water molecules [117]. In this regard, proton is defined as the formation of H_3_O^+^ species with water clusters that are transferred upon hydrogen bond breaking. In this way, protons are continuously jumping in the conduction path through water molecule protonation and deprotonation. Additionally, the vehicle mechanism is the proton migration by self-diffusion for proton carriers or protic species (H_3_O^+^, NH_4_^+^) [118, 119]. Moreover, the *E*_*a*_ involved in the Grotthuss mechanism is typically less than 0.4 eV since the energy to be dissipated for hydrogen bond breaking is about 2–3 kcal mol^−1^ (~ 0.11 eV). In contrast, the migration of larger ionic species typically requires greater energy provision in the vehicle mechanism, and therefore, the *E*_*a*_ for proton migration is higher than 0.4 eV. The effect factors and calculation process proton conductivity can be represented by Eq. 3.

3 $$\sigma = \frac{{ne^{2} \;D_{0} \;{\text{exp}}\left( {\frac{{\Delta S_{m} }}{k}} \right)}}{{{\text{kT}}}}{\text{exp}}\left( {\frac{{ - E_{a} }}{{{\text{kT}}}}} \right) \to \sigma = \frac{{\sigma _{0} }}{{{\text{kT}}}}{\text{exp}}\left( {\frac{{ - E_{a} }}{{{\text{kT}}}}} \right)$$

where $\sigma$ represents the proton conductivity (S cm^−1^), *n* represents the number of charge carriers, *e* represents the charge of the mobile ion, *D*_*0*_ represents a constant, *k* represents the Boltzmann constant, *T* represents the Kelvin temperature, $\Delta$*S*_*m*_ represents the motional entropy, ${\sigma }_{0}$ represents proportional to the centration of the proton carrier. During the proton conduction process, the charge (*e*) of the mobile ion can be considered constant, and the mobility of the proton is influenced by kinetic factors to some extent, as well as the variable concentration (*n*) of the proton. Therefore, factors that contribute to the improvement of proton conduction properties in MOFs can be deduced: (i) increasing the charge carriers; (ii) increasing the motion entropy; (iii) reducing activation energy; (iv) extending the proton conduction channel. The examples discussed subsequently also follow these factors for the enhancement of proton conduction.

## Proton Conduction Behavior in MOFs

**Fig. 6:**
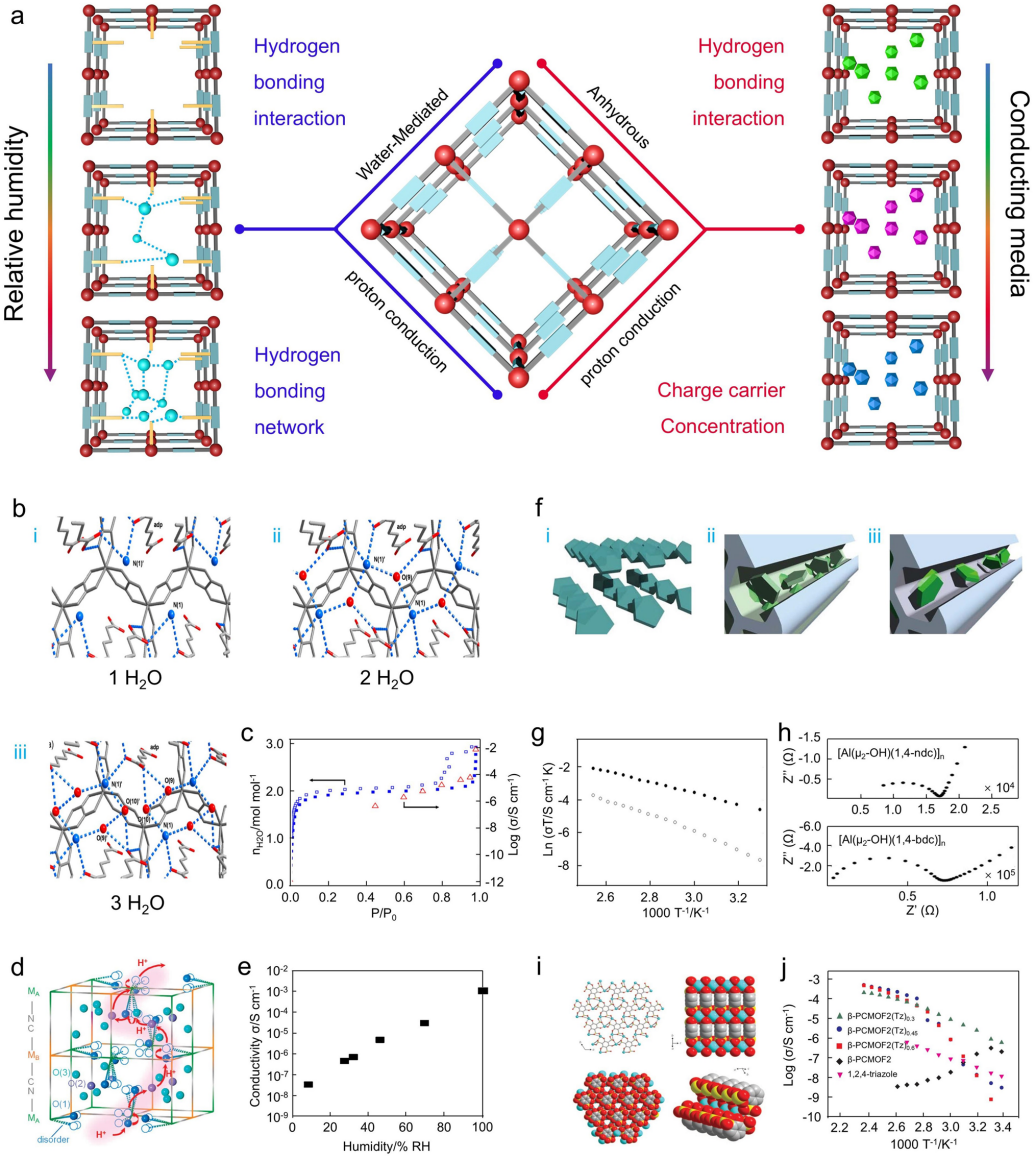
**a** Schematic representation of water-mediated and anhydrous proton conduction in MOFs. **b** Hydrogen bonding networks of (NH_4_)_2_(adp)[Zn_2_(ox)_3_]·H_2_O, (NH_4_)_2_(adp)[Zn_2_(ox)_3_]·2(H_2_O), and (NH_4_)_2_(adp)[Zn_2_(ox)_3_]·3(H_2_O). **c** The proton conductivity/adsorption isotherms. (Reproduced with permission from Ref. [125]. Copyright 2014, American Chemistry Society) **d, e** Proton conduction networks in M_A_[M_B_(CN)_6_]_2/3_·ZH_2_O and the proton conductivity of Co^II^[Cr^III^(CN)_6_]_2/3_·zH_2_O at 293 K. (Reproduced with permission from Ref. [126]. Copyright 2010, American Chemistry Society) **f** Imidazole accommodated in nanochannels of MOFs with/without active sites. **g, h** Proton conductivities and Nyquist plots of [Al(μ_2_-OH)(1,4-ndc)]_n_ and [Al(μ_2_-OH)(1,4-bdc)]_n_ under anhydrous conditions. (Reproduced with permission from Ref.[129]. Copyright 2009, Nature Publishing Group) **i** Single-crystal structure of β-PCMOF2. **j** Arrhenius plots of *β*-PCMOF2 and [*β*-PCMOF2(Tz)_x_] in anhydrous condition. (Reproduced with permission from Ref.[123]. Copyright 2009, Nature Publishing Group)

Indeed, the proton conduction properties of proton-conducting materials are closely related to water molecules, as water molecules can act as both proton carriers and proton donors. In general, water-mediated proton conduction mainly involves high humidity and low temperature (< 85 °C) conditions [120–122]. The anhydrous proton conduction generally represents relatively high operating temperatures (> 100 °C) [123, 124]. Therefore, the proton-conducting behavior in MOFs can be divided into two types (Fig. 6a): (i) water-mediated proton conduction; (ii) anhydrous proton conduction.

### Water-Mediated Proton Conduction

In water-mediated proton conduction, the hydrogen bonding interactions induced by water molecules have a crucial role effect in proton migration. To verify the intrinsic association between proton conductivity and proton conduction pathway, Sadakiyo and coworkers observed the trends of the hydrogen bonding network in proton-conducting MOFs ((NH_4_)_2_(adp)[Zn_2_(ox)_3_]·*n*H_2_O) under different humidity conditions (Fig. 6b) [125]. In brief, the hydrogen bonding network of (NH_4_)_2_(adp)[Zn_2_(ox)_3_]·*n*H_2_O consists of water molecules, ammonium ions (NH_4_^+^), and carboxylic acid groups of adipic acids (adp), where the water molecules and NH_4_^+^ are located between adp molecules. In this process, water molecules serve as a conductive medium that takes a critical role in the process of proton conduction and serves as a trigger for regulating proton conductivity by modifying the hydrogen bonding network through the adsorption/desorption process of water. Therefore, the proton conductivity of fully hydrated (NH_4_)_2_(adp)[Zn_2_(ox)_3_]·3H_2_O was 8.0 × 10^–3^ S cm^−1^ at 25 °C and 98% RH (Fig. 6c). Notably, the proton conductivity of (NH_4_)_2_(adp)[Zn_2_(ox)_3_]·*n*H_2_O is significantly influenced by the variation of humidity conditions as they affect the number of H_2_O molecules adsorbed by (NH_4_)_2_(adp)[Zn_2_(ox)_3_]. For example, the proton conductivity of (NH_4_)_2_(adp)[Zn_2_(ox)_3_]·3H_2_O is about 100 times higher than (NH_4_)_2_(adp)[Zn_2_(ox)_3_]·2H_2_O (7.0 × 10^–5^ S cm^−1^). For (NH_4_)_2_(adp)[Zn_2_(ox)_3_], the proton conductivity can be controlled from 10^–12^ to 10^–2^ S cm^−1^ by varying the number of guest water molecules, which directly verifies the correlation between the adsorbed water molecules and the proton conduction pathways, and corroborates the water-mediated proton conduction effect.

In addition, Ohkoshi and coworkers investigated the proton conduction properties of Prussian blue analogues under different environment conditions (Fig. 6d) [126]. The proton conductivity of two Prussian blue analogues (V[Cr(CN)_6_]_2/3_)·4.2H_2_O, Co[Cr(CN)_6_]_2/3_)·4.8H_2_O was 1.6 × 10^–3^ and 1.2 × 10^–3^ S cm^−1^ at 20 °C and 100% RH, respectively (Fig. 6e). In this regard, the proton transport takes place through the hydrogen bonding network of zeolite water molecules, so the proton conduction mechanism can be represented by Grotthuss mechanism. Moreover, the proton conductivity of Co[Cr(CN)_6_]_2/3_)·4.8H_2_O is significantly dependent on humidity, decreasing to 3.2 × 10^–8^ S cm^−1^ when the RH is reduced to 8%. For V[Cr(CN)_6_]_2/3_)·4.2H_2_O, the magnetic transition occurred at 310 k may lead to distortions in the hydrogen bonding network in its cubic network, which may have an impact on the proton conduction properties.

### Anhydrous Proton Conduction

Achieving efficient proton conduction at intermediate temperatures (100–200 °C) is a considerable challenge in fuel cell applications, which offers the following advantages: (i) faster kinetics of electrode reactions; (ii) higher conversion efficiency; (iii) lower CO poisoning [127, 128]. Therefore, the research on proton conduction in the anhydrous state has then received enormous attention. In this case, the critical factors for obtaining high proton conductivity are charge carrier concentration, structure, acidity, and alternative nonvolatile conducting medium (*e.g.*, triazole, imidazole, benzyl imidazole, ionic liquid, and strong hydrogen bonding interactions) (Fig. 6a). In this regard, Kitagawa and coworkers synthesized two imidazole-loaded Al-based MOFs ([Al(μ_2_-OH)(1,4-ndc)]_n_ and [Al(μ_2_-OH)(1,4-bdc)]_n_) and studied their proton conduction behavior in the anhydrous state for the first time (Fig. 6f) [129]. Indeed, the molecular mobility of the conducting medium is the key to achieving anhydrous high proton conductivity. Although Al-based MOFs have 1D pore channels with dimensions of about 8 Å bridged by hydroxyl groups, the surface potentials, pore shapes, and doped imidazole contents show large differences. Briefly, the spatial site resistance created by the benzene ring of the naphthalene ligand exposed in the pore direction in [Al(*μ*_2_-OH)(1,4-ndc)]_n_ negatively affects the interaction between the imidazole and μ_2_-OH bridged carboxylate around the metal center. In comparison, [Al(*μ*_2_-OH)(1,4-bdc)]_n_ has only one benzene ring in which the polar sites are prominent along the pore direction. That is, differences in the affinity of host–guest interactions can significantly affect the mobility of guest molecules and thus influence proton conductivity. Therefore, the imidazole-loaded [Al(*μ*_2_-OH)(1,4-ndc)]_n_ and [Al(*μ*_2_-OH)(1,4-bdc)]_n_ exhibited the proton conductivity of 2.2 × 10^–5^ and 1.0 × 10^–7^ S cm^−1^ at 120 °C, respectively (Fig. 6g, h).

Similarly, Shimizu and coworkers presented a crystalline Na-based sulfonate coordination material (*β*-PCMOF2) loaded with amphiphilic heterocycles (1H-1,2,4-triazole, Tz) for efficient proton conduction at 150 °C (Fig. 6i) [123]. The Na-based sulfonate MOFs have a honeycomb structure with 1D pores and exist in two regions, the *α*-phase in low temperature and *β*-phase in high temperature. The water molecules are substituted with Tz for proton conductivity by assembling Tz into *α*-phase and sequentially converting to *β*-phase under heating conditions. Therefore, the *β*-PCMOF2(Tz)_x_ exhibited the proton conductivities of 2.0 × 10^–4^ S cm^−1^ (x = 0.3), 5.0 × 10^–4^ S cm^−1^ (x = 0.45), and 4.0 × 10^–4^ S cm^−1^ (x = 0.6) at 150 °C, respectively (Fig. 6e). Moreover, the *E*_*a*_ of *β*-PCMOF2(Tz)_0.3_ was 0.51 eV at the temperature range of 23–150 °C, the *E*_*a*_ of *β*-PCMOF2(Tz)_0.45_ was 1.8 and 0.3 eV between 50–90 and 90–150 °C, the *E*_*a*_ of *β*-PCMOF2(Tz)_0.6_ was 1.87 and 0.56 eV between 23–80 and 80–150 °C (Fig. 6j).

In contrast to water-mediated proton conduction, the maximum proton conductivity of MOFs under anhydrous conditions is about 10^–3^ S cm^−1^. As we mentioned above, both water-mediated and anhydrous proton conduction are strongly dependent on the nature of the hydrogen bonds formed. Indeed, in both proton conduction behaviors, improving the mobility of active protons and facilitating the generation of hydrogen bonding networks within the pores are crucial factors for improving the proton conduction performance.

## Constructive Strategies for Imparting Proton Conductivity to MOFs

**Fig. 7:**
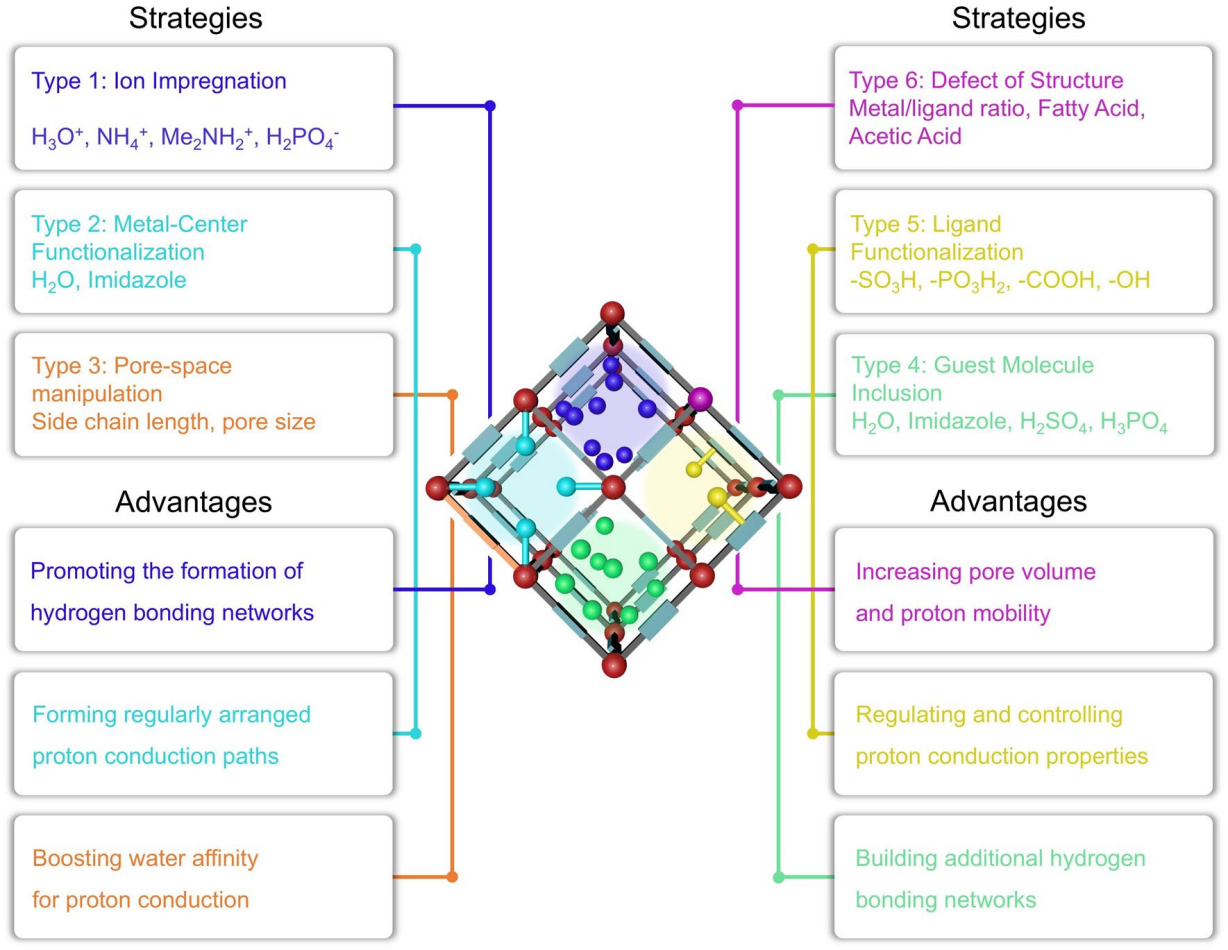
Schematic illustration of strategies to impart proton conductivity to MOFs and their advantages

Currently, proton-conducting MOFs can be constructed by various design principles and methods to enhance the acidic concentration of carriers and the mobility of efficient conduction pathways, including charged frameworks construction, ligand functionalization, metal-center manipulation, defective engineering, guest molecule incorporation, and pore-space manipulation (Fig. 7). In this section, several representative strategies for proton-conducting MOFs are discussed and analyzed in depth, such as (i) charged frameworks construction; (ii) ligand functionalization; (iii) metal-center manipulation; (iv) defective engineering; (v) guest molecule incorporation; (vi) pore-space manipulation. Moreover, these construction strategies mainly rely on the design principles of bottom-up design and post-synthesis modification [130]. The design principle of post-synthetic modification has received more attention and utilization due to the increased possibilities. Specifically, the covalent post-modification is the formation of new covalent bonds with components of MOFs through the use of modifiers, which can effectively introduce a variety of chemical groups into MOFs, thereby altering the pore size of MOFs without changing the basic structure of MOFs. Coordination post-modification refers to the removal of end-coordinated solvent molecules by heating in some MOFs with multiple coordination bonds to create open metal sites, and these metal vacancies can be modified using other ligands to change the pore size of MOFs. For example, direct immersion of MOFs in solutions containing other ligands may also directly displace solvent molecules, generating coordination vacancies that can be coordinated with other modifiers, thus modifying the pore size of MOFs. Moreover, some ligands in MOFs can also be eliminated by ligand changes in the metal clusters, while these vacancies can be restored by the introduction of other ligands that can change the pore size of MOFs.

### Charged Frameworks Construction

Through the topological connection of metal cationic and organic ligand anions, most MOFs typically exhibit a neutral framework. In general, the charged networks of MOFs can be pre-designed by rationally combining organic ligands, metal ions, and organic solvents [52, 131]. In this case, the charged MOFs will impregnate cationic or anionic species as counterbalance ions in their pore space to achieve a charge balance through inherently electrostatic interactions. In anionic frameworks, amide solvents such as N,N-diethylformamide (DEF), N,N-dimethylacetamide (DMA), and N,N-dimethylformamide (DMF) can be decomposed by hydrolysis under solvothermal conditions to [NH_2_Et_2_]^+^ or [NH_2_Me_2_]^+^ cations and act as counterions to obtain the proton conductivities from 10^–4^ to 10^–2^ S cm^−1^ at low RH conditions. In this regard, several proton species in the anionic framework (*e.g.*, NH_2_Me_2_^+^, NH_2_Et_2_^+^, and NH_4_^+^) can be involved in proton diffusion via molecular diffusion or proton donation in conjunction with formed hydrogen bonds. In cationic frameworks, halide counterions have attracted enormous attention because they are expected to be novel positively charged frameworks for MOFs, increasing the proton conductivity to 10^–4^–10^–2^ S cm^−1^.

**Fig. 8:**
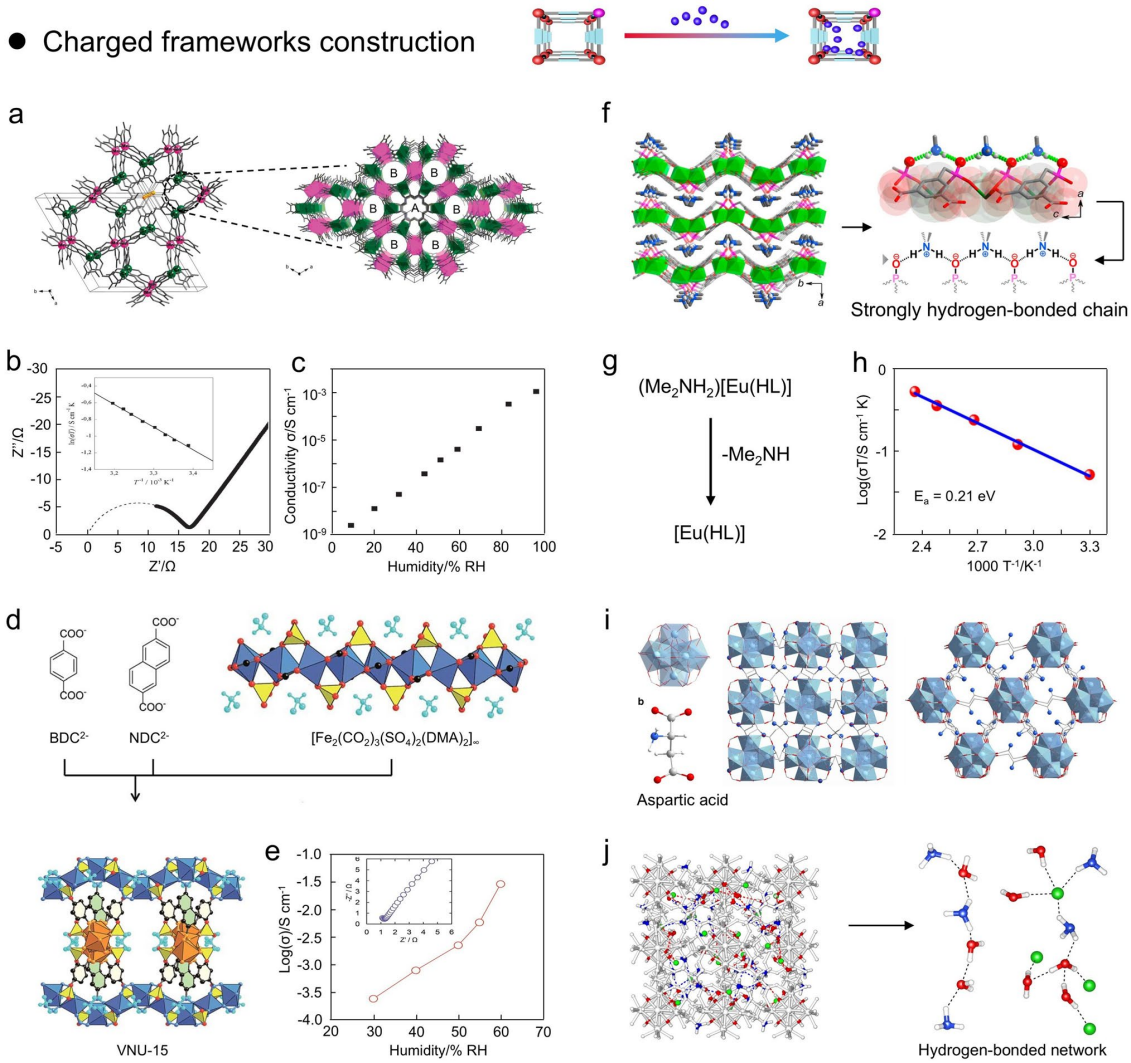
**a** Perspective view and crystal structure of (NH_4_)_4_[MnCr_2_(ox)_6_]·4H_2_O. **b, c** Impedance and proton conductivity of (NH_4_)_4_[MnCr_2_(ox)_6_]·4H_2_O at 295 K and 96% RH. (Reproduced with permission from Ref. [132]. (Copyright 2011, American Chemistry Society) **d** Crystal structure of VNU-15 and preparation process from BDC^2−^, NDC^2−^, and [Fe_2_(CO_2_)_3_(SO_4_)_2_(DMA)_2_]_∞_. **e** Proton conductivity in VNU-15 at 95 °C. (Reproduced with permission from Ref. [133]. Copyright 2016, Royal Society of Chemistry) **f** Schematic representation of the periodic arrangement of the counter cations (Me_2_NH_2_)^+^ in the intercalation of (Me_2_NH_2_)[Eu(L)] and its hydrogen bond formation with uncoordinated oxygen. **g** Proton conduction from anionic structure to neutral structure by removing Me_2_NH molecules. **h** Arrhenius plot of (Me_2_NH_2_)[Eu(L)] from 30 to 150 °C at anhydrous conditions. (Reproduced with permission from Ref. [134]. Copyright 2017, American Chemistry Society) **i** The crystal structure of MIP-202(Zr). **j** Schematic representation of multiple hydrogen bonding networks in MIP-202(Zr). (Reproduced with permission from Ref. [135]. Copyright 2018, Nature Publishing Group)

From the intrinsic proton conduction properties, Verdaguer and coworkers proposed an oxalate-based anionic MOFs ((NH_4_)_4_[MnCr_2_(ox)_6_]_3_·4H_2_O) with high proton conductivity (Fig. 8a) [132]. The functionalized nanochannels of oxalate bimetallic compounds incorporate ammonium ions and water molecules and are also divided into A and B with different sizes of 5.23 and 7.52 Å, respectively. The ammonium ions are situated near the oxalate ligand of channel A and are used for charge compensation. Meanwhile, the terminal oxalate and ammonium ions form hydrogen bonds at a distance of 2.795–2.960 Å, and these hydrogen bonds are connected to the central water channel. Therefore, owing to the existence of these multi-dimensional hydrogen bonding networks, the synthesized (NH_4_)_4_[MnCr_2_(ox)_6_]_3_·4H_2_O exhibited high proton conductivities of 1.1 × 10^–3^ and 1.7 × 10^–3^ S cm^−1^ at 295 and 313 K under 96% RH, respectively (Fig. 8b and c). Along a similar research path, Furukawa and coworkers report an anionic Fe-based MOFs (VNU-15) with high proton conductivity at low RH (Fig. 8d) [133]. In brief, VNU-15 was fabricated by heating H_2_NDC, H_2_BDC, CuCl_2_, and FeSO_4_, where 9,10-anthraquinone and CuCl_2_ acted as redox agents to generate DMA. Moreover, the metal ions in [Fe_2_(CO_2_)_3_(SO_4_)_2_(DMA)_2_] were connected by SO_4_^2−^, and then two ligands interconnected the metal chains. Due to the integration of sulfate ligands and hydrogen-bonded dimethylammonium ions in the nanopores of VNU-15, the proton conductivity can achieve 2.38 × 10^–4^ S cm^−1^ at 95 °C and 30% RH and 2.9 × 10^–2^ S cm^−1^ at 95 °C and 60% RH, respectively (Fig. 8e). Indeed, the hydrogen bonding network generated by the sulfate bridges in [Fe_2_(CO_2_)_3_(SO_4_)_2_(DMA)_2_] with the DMA cations is critical to high proton conductivity of the anionic VNU-15. Similarly, Zang and coworkers reported a charged Eu proton-conducting MOFs, (Me_2_NH_2_)[Eu(L)] (Fig. 8f) [134]. The compound was composed of a stratified anionic structure [Eu(L)]^−^ with counter cations (Me_2_NH_2_)^+^ embedded between layers, engaging in interactions with neighboring uncoordinated oxygen atoms, thereby creating a highly interconnected hydrogen-bonded oriented parallel to the c-axis (Fig. 8g). Thus, protons can migrate efficiently along these chains, allowing (Me_2_NH_2_)[Eu(L)] to achieve an anhydrous conductivity of 1.25 × 10^–3^ S cm^−1^ at 150 °C (*E*_*a*_ = 0.21 eV) and water-assisted proton conductivity of 3.76 × 10^–3^ S cm^−1^ for compacted crystal particles at 100 °C and 98% RH (*E*_*a*_ = 0.72 eV) (Fig. 8h). This study showed the unique proton transfers and vibrations between acid–base pairs in hydrogen-bonded chains are generated by electrostatic interactions.

Additionally, Wang and coworkers presented cationic Zr-based MOFs (MIP-202) constructed from natural *α*-amino acids with exceptional and stable proton conduction properties (Fig. 8i) [135]. In sharp contrast to the synthesis of other MOFs, MIP-22 was prepared by heating an aqueous mixture of L-aspartic acid and ZrCl_4_ under ambient pressure with reflux for several hours. Notably, MIP-202 was synthesized only at high concentrations of the reactants, which may be attributed to the strongly acidic conditions of Zr^4+^ that affect the presented conformation of the amino-containing linker. In other words, amino acids with more basic -NH_2_ groups will undergo complete protonation upon the existence of strongly acidic metal cations, resulting in the creation of zwitterionic ligands, which in turn affects the ability of amino acids to coordinate with high-valent metal (Fig. 8j). The presence of Brønsted acid, superior stability, and porous structure endow MIP-202 with the potential to become high-performance proton conductors. Therefore, MIP-202 showed high and stable proton conductivity of 1.1 × 10^–2^ S cm^−1^ at 363 K and 95% RH. Moreover, the roles of NH_3_^+^ and Cl^−^ during the generation of hydrogen bonding networks were further revealed by Monte Carlo molecular simulation calculations. The results showed the H_2_O molecules were arranged on the framework of MIP-202 via the interaction of their oxygen atoms with the protons of NH_3_^+^, which could be demonstrated by the short average distances between the two atom pairs (1.8 Å) and the protons of Zr oxygen cluster (1.9 Å) (Fig. 8j).

### Ligand Functionalization

**Fig. 9:**
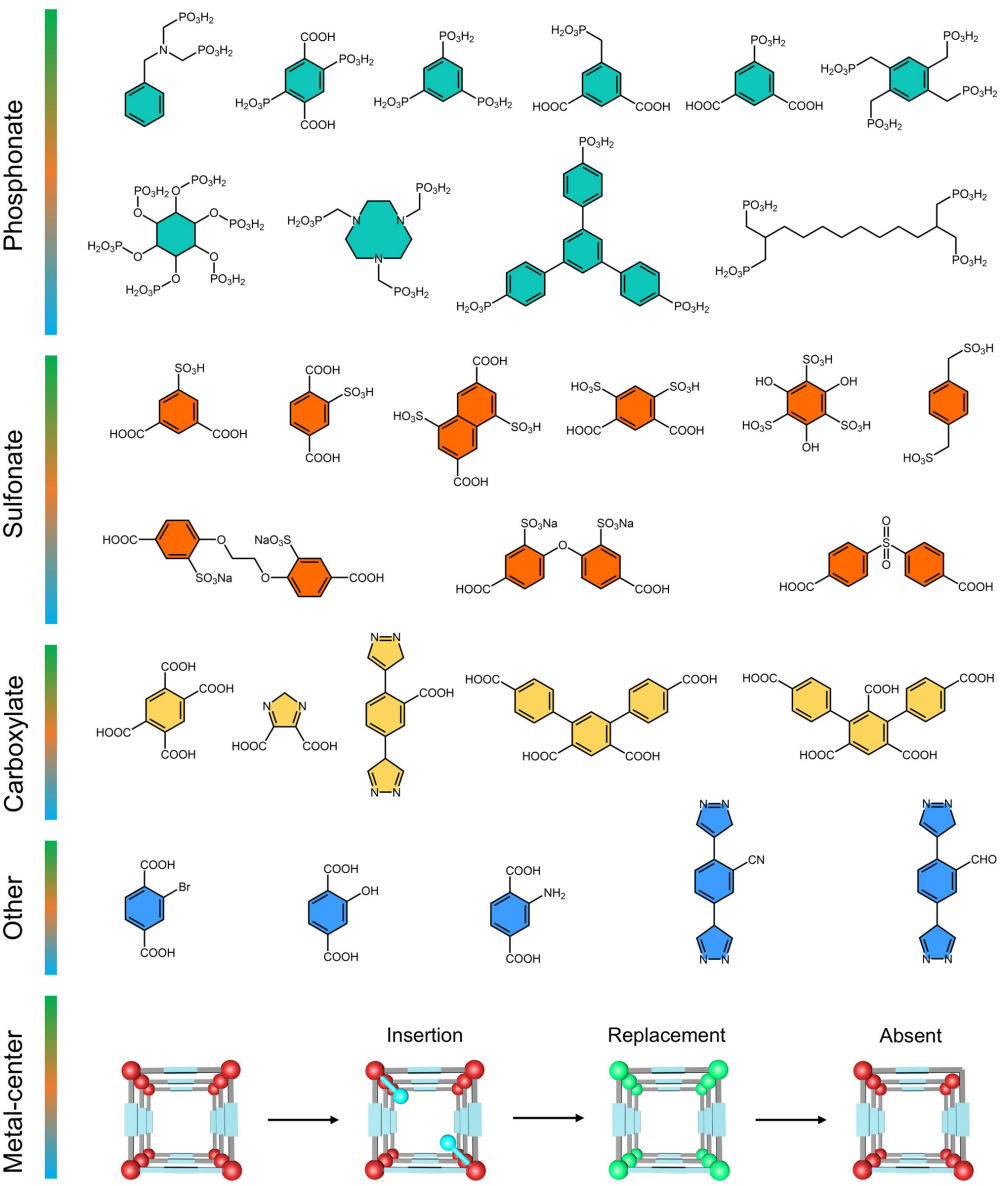
Schematic illustration of representative additional functional groups in proton-conducting MOF ligands and the manipulation of metal centers

The tunability and designability of organic linkers present amazing opportunities for performance regulation of MOF materials. The incorporation of various functional groups or manipulation of metal atoms can change the nature of the pore and surface environments of MOFs. In general, functionalized modification of the ligands of MOFs was performed by employing the strategy of predesign and post-synthesis modification [136–138]. In terms of ligand functionalization, the incorporation of uncoordinated or partially coordinated acid functional groups (*e.g.*, –PO_3_H_2_, –SO_3_H, –COOH, and others) into the ligand is a common approach (Fig. 9). In this regard, the addition of acidic functional groups is favorable to increasing the carrier concentration of proton-conducting MOFs. Moreover, other functional groups (*e.g.*, hydroxyl groups, amines) can gain additional proton hopping sites via hydrogen bonding networks. Particularly, the manipulation of metal centers in MOFs by ligand insertion, metal substitution, or metal defects can significantly affect the properties of MOFs (Sect. 5.3).

#### Predesigned Ligand Functionalization

**Fig. 10:**
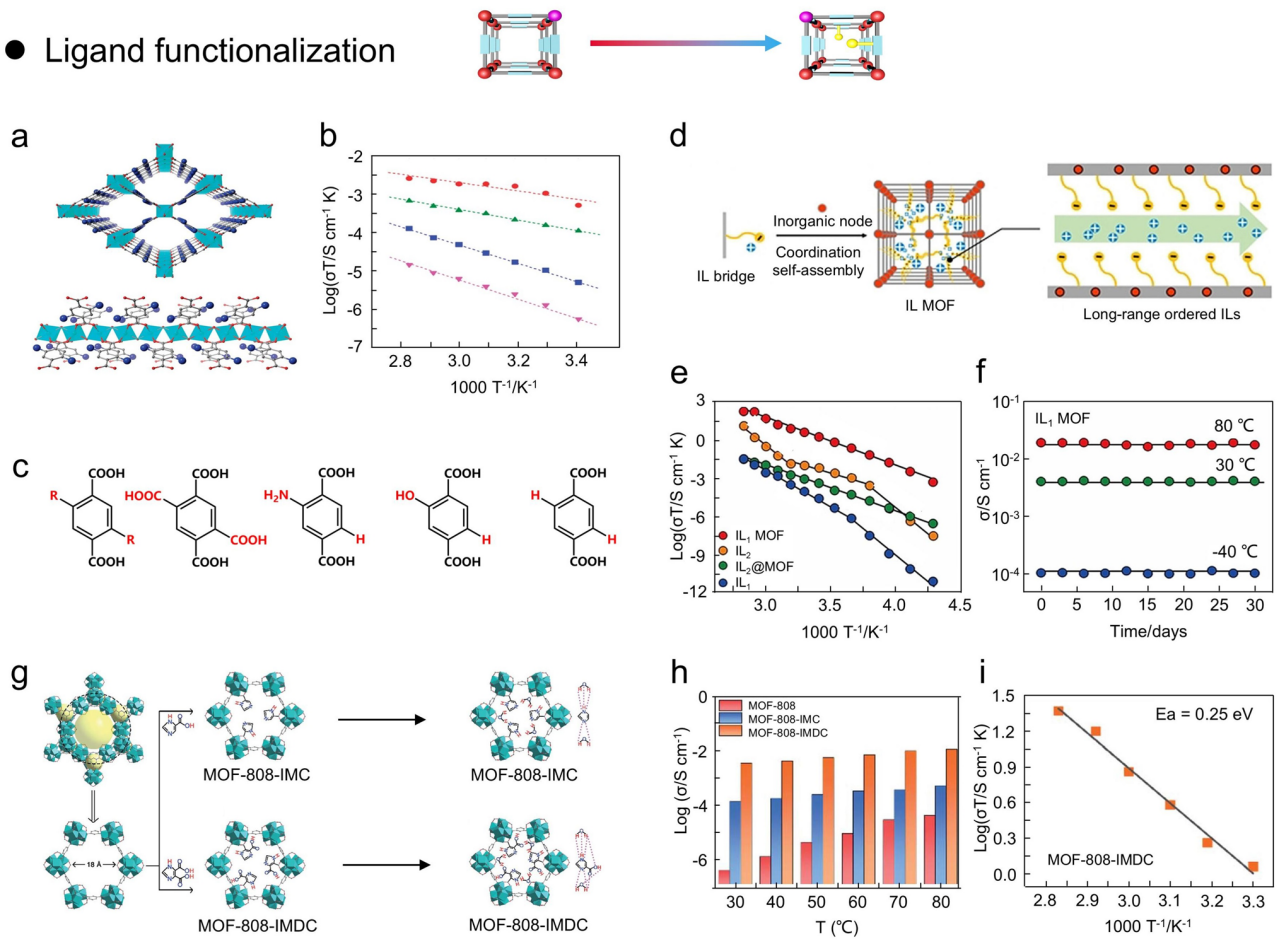
**a-c** Ligand functionalization in MIL-53 (M) and Arrhenius plots of different ligands under 95% RH. (Reproduced with permission from Ref. [139]. Copyright 2011, American Chemistry Society) **d** Design strategies of crystalline ILMOF with long-range ordered framework. **e, f** Proton conductivities of IL_1_MOF, IL_2_@MOF, IL_1_, IL_2_, and long-term stability of IL_1_MOF. (Reproduced with permission from Ref. [140]. Copyright 2021, Wiley–VCH) **g** Synthesis of MOF-808-IMC and MOF-808-IMDC. **h** Proton conductivities of MOF-808, MOF-808-IMC, and MOF-808-IMDC at 98% RH. **i** Arrhenius plot of MOF-808-IMDC under 98% RH (Reproduced with permission from Ref. [146]. Copyright 2022, Royal Society of Chemistry)

A representative example of the effect of ligand functionalization on the proton conductivity in MOFs (MIL-53) was reported by Kitagawa and coworkers (Fig. 10a) [139]. The MIL-53 was fabricated by the 1,4-benzenedicarboxylate with different functional groups (-COOH, -NH_2_, -OH) (Fig. 10b). The hydroxyl ligands bridge the metal units to form 1D chains, while isophthalic acids interconnect the metal chains to create 3D structures with high chemical stability and structural flexibility. Notably, the functional groups on ligands protrude toward the pores, and the differences in functional groups significantly affect the acidity (*pK*_*a*_) of the MOFs (3.62 for -COOH, 4.08 for –OH, 4.19 for –H, and 4.74 for –NH_2_). The proton conductivities of four MOFs are 2.0 × 10^–6^ (–COOH), 4.2 × 10^–7^ (–OH), 2.3 × 10^–9^ (–NH_2_), and 2.3 × 10^–8^ S cm^−1^ (–H), respectively. Moreover, the corresponding *E*_*a*_ are 0.21, 0.27, 0.45, and 0.47 eV, respectively (Fig. 10c). That is, the acidity of MOFs correlates well with proton conductivity (–COOH > –OH > –H > –NH_2_). MIL-53(Al)-OH and MIL-53(Al)-NH_2_ have been reported to exhibit different water adsorption at 25 °C [139]. Briefly, MIL-53(Al)-OH has a proton conductivity of about 10^–6^ S cm^−1^ at 25 °C and 95% RH, while MIL-53(Al)-NH_2_ is about 10^–9^ S  cm^−1^. Therefore, the proton conduction mechanism in MOF pores with acidic functional groups can be illustrated below: (i) The acidic functional group within MOFs spontaneously transfers protons to H_2_O molecules via a hydration process, resulting in the formation of a H_3_O^+^–H_2_O acid–base conjugated systems within the MOF channels; (ii) these acid–base conjugated systems are interconnected to form continuous proton conduction channels that facilitate proton transport.

In addition, Xu and coworkers constructed crystalline IL_1_MOFs (UiO-67) with high proton conductivity properties by designing ionic liquid (IL) bridged ligands for coordination self-assembly with metal clusters (Fig. 10d) [140]. Notably, IL_1_MOF possesses a distinctive structure in which the IL-linked ligand is arranged on an ordered framework, which allows IL_1_MOF to overcome the typical limitation that solid ILs have lower proton conductivity than their corresponding bulk ILs. In particular, the proton conductivity of IL_1_MOF is higher than that of IL monomer by 2–4 orders of magnitude over a wide temperature range. Notably, conventional studies have impregnated ILs into MOF to form ILs@MOF composites with proton conductivity. However, the ILs@MOF composites cannot form crystalline IL networks in MOFs due to the difficulty of precisely controlling the weak interactions between ILs and MOF frameworks, and the ILs were in an atactic state, so the proton conductivity of ILs@MOF composites was still much lower than bulk ILs (Fig. 10e) [141, 142]. Briefly, IL_1_ was synthesized by covalently bonding the composite part ([-MIMS][MSA]) of IL_2_ to 4,4′-biphenyldicarboxylate acid (H_2_BPDC). IL_2_ is a complex of methanesulfonic acid (MSA) and 1-(1-ethyl-3-imidazolio)-propane-3-sulfonate (EIMS). The MIMS-MOF was formed by the reaction of H_2_BPDC MIMS with ZrCl_4_ under solvothermal conditions. Thus, the sulfonic acid groups grafted to MIMS on MIMS-MOF can be dissociated from MSA by Brønsted acid–base buffering, resulting in the construction of a novel ionic liquid with a rigid lattice framework. Noteworthy, the proton conductivity of IL1MOF can reach 2.87 × 10^–3^ S cm^−1^ at 20 °C and 98% RH, which is 2 orders of magnitude higher than ILs. Further, the proton conductivity stability tests were performed by immersing IL_1_MOF and IL_2_MOF in deionized water for 20 min. The proton conductivity of IL2@MOF was reduced by 4 orders of magnitude, whereas IL1MOF showed superior test stability (Fig. 10f).

#### Post-Synthetic Ligand Functionalization

Apart from the synthesis of proton-conducting MOFs with pre-designed ligands, the strategy of post-synthesis functionalization of ligands can also be employed to efficiently build novel proton-conducting MOFs [143–145]. Briefly, the ideal requirement for post-synthetic modification of ligands in MOFs is to maintain overall structural integrity, especially when modification of ligand functional groups is involved. In this regard, the new functional groups can be used as adsorption sites for various target molecules, mainly achieved without changing the structure of the parent framework [130]. For proton-conducting MOFs, the functional groups of strong Brønsted acids (*e.g.*, –SO_3_H, –PO_3_H_2_, and –COOH) can bind to numerous proton donors to create a sequential hydrogen bonding network, thereby facilitating the efficient proton conduction. Indeed, the integration of acidic groups into MOFs necessitates the use of precursors with multiple acidic sites or other substances with higher ligand affinity for metal centers. In particular, the acidic groups will disturb the interactions between neighboring groups and metal sites when the precursor is very densely populated with acidic groups, affecting the synthesis of MOFs to some extent. In this regard, Zeng and coworkers synthesized MOF-808-IMC and MOF-808-IMDC with ultra-high proton conductivity by employing 1*H*-imidazole-4-carboxylic acid (IMC) and 4,5-imidazoledicarboxylic acid (IMDC) to replace the formate and hydroxide groups aligned to Zr in MOF-808 (Fig. 10g) [146]. Briefly, the IMC/IMDC and MOF-808 were mixed in an aqueous solution and stirred at 90 °C for 48 h. The precipitates were subsequently collected to obtain MOF-808-IMC and MOF-808-IMDC. Notably, MOF-808 is chemically and thermally stable in both aqueous and acidic environments, whereas hydroxides, water molecules, and formates in coordination with Zr can be substituted with other more coordinating groups. For IMC and IMDC containing imidazole and carboxylic acid groups, the imidazole can facilitate proton conduction, while the carboxylic acid groups can substitute hydroxides, water molecules, and formates on the MOF-808 framework. Therefore, the proton conductivities of MOF-808-IMC (5.04 × 10^–4^ S cm^−1^) and MOF-808-IMDC (1.11 × 10^–2^ S cm^−1^) are much higher than the original MOF-808 (4.21 × 10^–5^ S cm^−1^) at 80 °C and 98% RH (Fig. 10h). The *E*_*a*_ of MOF-808, MOF-808-IMC, and MOF-808-IMDC are 0.9, 0.27, and 0.25 eV, respectively, which shows the proton conduction mechanism of MOF-808 is vehicle mechanism, MOF-808-IMC and MOF-808-IMDC is Grotthuss mechanism (Fig. 10i).

Moreover, Hong and coworkers employed a post-synthesis oxidation strategy to convert UiO-66(SH)_2_ to UiO-66(SO_3_H)_2_ with high stability and proton conductivity [147]. Notably, UiO-66 type MOF possesses exceptional water stability because of the existence of large metal clusters formed by the coordination of multiple ligands. To transform UiO-66(SH)_2_ to UiO-66(SO_3_H)_2_, it was necessary to perform a 1 h oxidation reaction with H_2_O_2_ solution followed by a continuous protonation reaction with H_2_SO_4_ solution. As expected, UiO-66(SH)_2_ and UiO-66(SO_3_H)_2_ exhibited the superior proton conductivities of 6.3 × 10^–6^ and 1.4 × 10^–2^ S cm^−1^ at 25 °C and 90% RH. At 80 °C and 90% RH, the proton conductivity of UiO-66(SO_3_H)_2_ increased to 8.4 × 10^–2^ S cm^−1^ with a low *E*_*a*_ value. The remarkable proton conductivity of UiO-66(SO_3_H)_2_ is attributed to the presence of Brønsted acid sites (–SO_3_H) on organic linkers. These acidic groups can promote the adsorption of water molecules preferentially into the confined space of MOFs, leading to the organization of hydrophilic structural domains and the establishment of proton conduction pathway comparable to that of Nafion. Notably, the structural stability of the MOF host material is a precondition to prevent chemical decomposition during the implementation the post-synthetic modifications. That is, this stringent performance requirement limits the generality of the post-synthetic modification strategy for application in MOFs to some extent.

### Metal-Center Manipulation

According to the principle of coordination chemistry, the geometry of the ligand and the coordination number of the metal ion are the critical factors affecting the structure and properties of MOFs [148–151]. Notably, the coordination solvents (*e.g.*, MeOH, EtOH, DEF, DMF) contained in the metal clusters can be eliminated by vacuum/heating or supercritical CO_2_ drying, resulting in the creation of free coordination sites known as “open metal sites” during the synthesis of MOFs. These open metal sites provide strong active sites for adsorption or catalytic reactions with guest molecules. For proton-conducting MOFs, there are two metal-center manipulation strategies: (i) insertion of functional organic molecules; (ii) metal-center replacement.

#### Coordinative Insertion

**Fig. 11:**
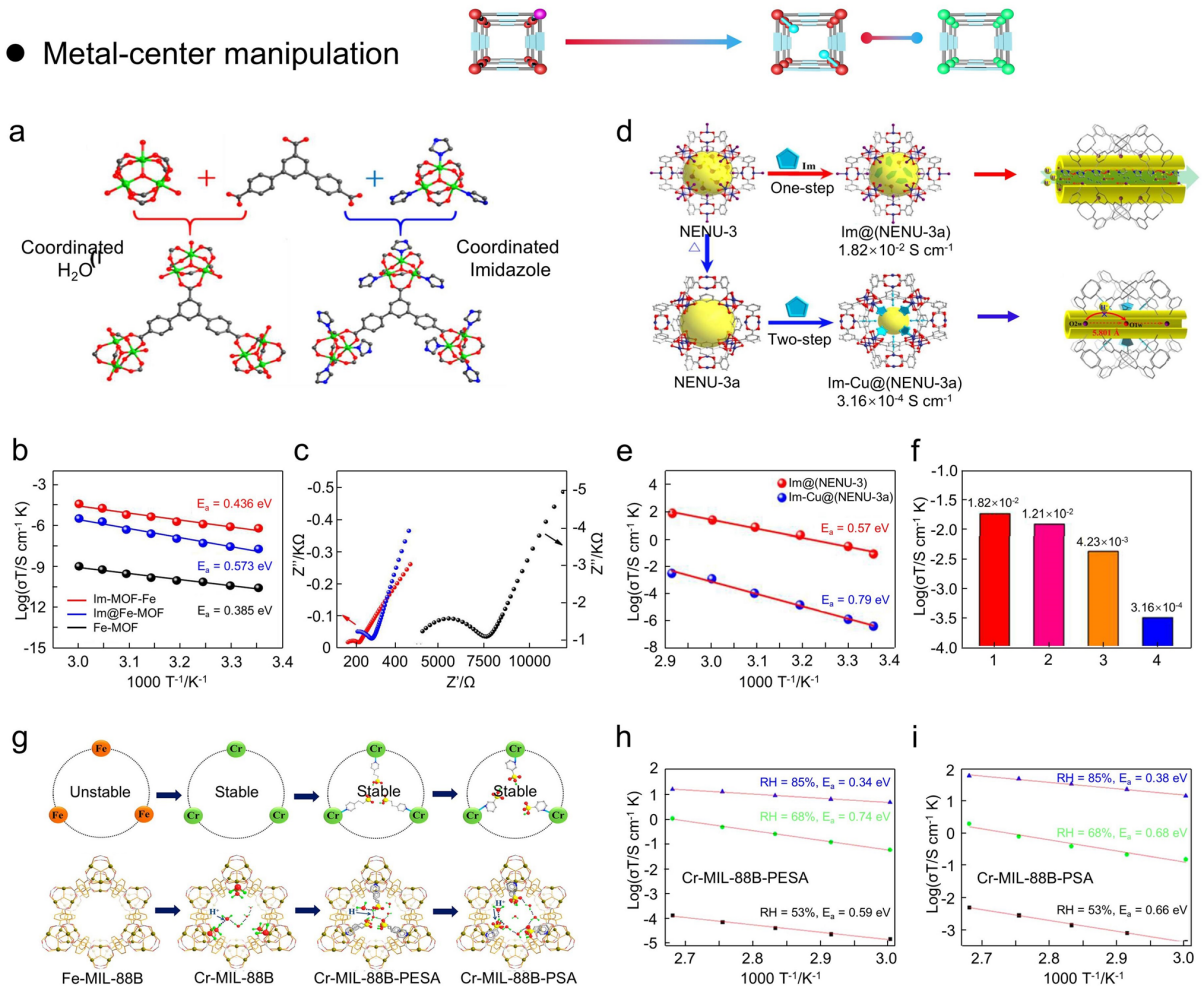
**a** Ligand–metal cluster attachment patterns in Fe-MOF and Im-Fe-MOF. **b, c** Arrhenius plots and impedance spectrum of Im-Fe-MOF, Im@Fe-MOF, and Fe-MOF under 98% RH. (Reproduced with permission from Ref. [153]. Copyright 2017, American Chemistry Society). **d** Fabricate process of Im@(NENU-3) and Im-Cu@(NENU-3a). **e** Arrhenius plots of Im@(NENU-3) and Im-Cu@(NENU-3a). **f** Proton conductivity of Im@(NENU-3) (1), Im-Fe-MOF (2), Im@Fe-MOF (3), and Im-Cu@(NENU-3a) (4). (Reproduced with permission from Ref. [154]. Copyright 2017, American Chemistry Society) **g** Schematic diagram of the substitution and modification of Fe metal atoms in Fe-based MIL-88B. **h, i** Arrhenius plots of Cr-MIL-88B-PESA and Cr-MIL-88B-PSA (Reproduced with permission from Ref. [156]. Copyright 2020, American Chemistry Society)

Imidazole molecules (Ims) are widely doped into porous materials to enhance proton conduction properties [152, 153]. To investigate the different arrangement behaviors of imidazole in MOFs affecting the proton conduction mechanism, Zhou and coworkers designed and prepared three compounds (Fe-MOF, Im-Fe-MOF, Im@Fe-MOF) with the same Fe-based MOF framework (Fig. 11a) [153]. Im@Fe-MOF represents Ims physically adsorbed in Fe-MOF nanopores, and Im-Fe-MOF represents Ims coordinated to metal centers of Fe-MOFs. The proton conductivity analysis shows that the liganded Ims in Im-Fe-MOF significantly facilitate the proton conduction than the physically absorbed Ims, and the proton conductivity of Im-Fe-MOF is about two orders of magnitude higher than Im@Fe-MOF at 25 °C. Specifically, the proton conductivities of Fe-MOF, Im-Fe-MOF, and Im@Fe-MOF were 2.56 × 10^–5^, 2.06 × 10^–3^, and 8.41 × 10^–5^ S cm^−1^ at 25 °C and 98% RH, respectively. Meanwhile, the *E*_*a*_ values of Fe-MOF, Im-Fe-MOF, and Im@Fe-MOF were 0.385, 0.436, and 0.573 eV at 98% RH, respectively (Fig. 11b, c). In contrast to Fe-MOF, the pores of Im@Fe-MOF were dominated by disorganized Ims along with lower water absorption, which could hinder the establishment of a continuous hydrogen bonding network. For Im-Fe-MOF, it has lower water absorption capacity and narrower nanochannels because the coordination occupies regularly arranged Ims. That is, the Ims immobilized by ligand bonds and regularly arranged in the framework are more conducive to the formation of proton conduction channels under humidity conditions than the disordered distribution of Ims.

Similarly, Chen and coworkers identified a MOF material (NENU-3) that contains free Ims with higher proton conductivity than samples containing bound Ims (Fig. 11d) [154]. NENU-3 was selected as the host for the encapsulated Ims because of the improved thermal and chemical stability of polyoxometalate anions into the HKUST-115 type framework. The axial sites of the Cu paddlewheel structure of NENU-3 were first dominated by water and later substituted by Ims through coordination modification (Im-Cu@NENU-3a). Moreover, the weight percentage of Ims in Im@(NUNE-3) was found to be 14.5 wt% by thermogravimetric analysis, which corresponds to 43.5 Ims per crystal cell, and higher than 36.7 Ims in the cells of Im-Cu@NENU-3a. Therefore, Im@(NUNE-3) exhibits the proton conductivity of 1.82 × 10^–2^ S cm^−1^ at 70 °C and 90% RH, which is higher than Im-Cu@NENU-3a by about two orders of magnitude. Indeed, Im@(NUNE-3) exhibits higher proton conductivity and lower *E*_*a*_ values mainly because the hydrogen bonding network generated between a high concentration of free Ims and water molecules, which constructs a fast and efficient proton transfer pathway and Grotthuss proton conduction mechanism (Fig. 11e, f).

In addition, Kitagawa and coworkers achieved stable ultra-high proton conductivity by inserting solvent-free-coordinative urea into MOF-74(Ni/Mg) without acidic moiety [155]. MOF-74 has a stable structure and numerous open metal sites, which is favorable for post-synthetic modification. To prevent moisture and solvent coordination in the open metal sites, the coordination urea insertion reaction was carried out by a solvent-free reaction at the urea melting point. Four compounds with different ligand urea insertions were fabricated: MOF-74(Ni)-H_2_O (0% urea), MOF-74(Ni)-H_2_O (8.5% urea), MOF-74(Ni)-H_2_O (100% urea), MOF-74(Mg)-H_2_O (100% urea). The porosity and pore size of MOF-74 decreased with increasing urea content. Notably, when the relative humidity was elevated to 95%, there was a significant increase in the proton conductivity values of MOF-74(Mg)-H_2_O (100% urea) and MOF-74(Ni)-H_2_O (100% urea), which were 2.64 × 10^–2^ and 6.19 × 10^–4^ S cm^−1^, respectively. Moreover, the *E*_*a*_ of MOF-74(Mg)-urea increased up to 1.16 eV at 70% RH, and decreased up to 0.37 eV at 95% RH, indicating a change in the proton conduction mechanism. Briefly, protons migrate mainly by molecular diffusion mechanism under low humidity conditions (< 70% RH), while the ligand urea serves as barrier though spatial perturbation. In particular, the regulated void space between confined water and polarized urea allows for persistent high proton conductivity through unique molecular dynamics in the absence of strongly acidic molecules in either framework of the guest.

#### Metal-Center Replacement

The replacement of metal-center involves the breaking of coordinate bonds between the organic branched chains and the metal ions, as well as the formation of new bonds with the incoming metal ions. Indeed, the replacement of metal-center depends on many factors: (i) the coordination preferences and valence of the incoming metal ions; (ii) the stability of metal ions to be exchanged; (iii) the solvents used in the substitution process; (iv) the chemical stability of the resulting MOFs. Indeed, only partial substitution of the metal ions has been observed in most substitution reactions at the metal ions. That is, it is difficult to achieve total replacement of the metal center in MOFs.

The structural stability of the MOF host is a precondition for preventing chemical decomposition during the implementation of the modification. From the perspective of the MOF metal centers, enhancing the connection between metal centers and ligands can effectively improve the stability of MOFs. In this regard, Zang and coworkers presented an improvement in the proton conductivity of Fe-MIL-88B by replacing the Fe^3+^ with Cr^3+^ followed by ligand intercalation of 3-pyridine-sulfonic acid (PSA) and 2-(4-pyridyl)ethane sulfonic acid (PESA) (Fig. 11g) [156]. Fe-MIL-88 is constructed from 1,4-benzendicarboxylic acid and Fe(III) trimer in a hexagonal channel along the c-axis. However, Fe-MIL-88B has weaker structural stability due to weaker Fe–O bonds. Cr-MIL-88B, Cr-MIL-88B-PESA, Cr-MIL-88B-PSA were synthesized by controlling the metal ions and coordination of organic molecules during the synthesis step. Notably, the water absorption of Cr-MIL-88B-PSA and Cr-MIL-88B-PESA was higher than Cr-MIL-88B because of the existence of hydrophilic sulfonic acid groups in the channels of Cr-MIL-88B-PSA and Cr-MIL-88B-PESA. Therefore, Cr-MIL-88B, Cr-MIL-88B-PESA, and Cr-MIL-88B-PSA showed proton conductivities of 6.0 × 10^–3^, 4.5 × 10^–2^, and 1.58 × 10^–1^ S cm^−1^ at 100 °C and 85% RH, respectively. In this regard, the possible coordinated water molecules and hydroxyl groups in Cr-MIL-88B would contribute the proton conduction. For Cr-MIL-88B-PSA and Cr-MIL-88B-PESA, the doped SO_3_H group both acts as an effective proton donor to improve the proton concentration and allow more water molecules to enter the nanopores of MOF to form a rich hydrogen bonding network. Moreover, the *E*_*a*_ of Cr-MIL-88B, Cr-MIL-88B-PESA, Cr-MIL-88B-PSA were 0.59, 0.34, and 0.38 eV at 85% RH, respectively (Fig. 11h, i), which suggest a Grotthuss mechanism for proton conduction in Cr-MIL-88B-PESA and Cr-MIL-88B-PSA, and vehicle mechanism for Cr-MIL-88B.

### Defective Engineering

In general, the extrinsic or intrinsic defects in solid porous materials play a definite role in their physicochemical properties [157–160]. As we mentioned above, MOF materials are typically synthesized by connecting organic ligands to inorganic nodes, such as metal or metal oxide clusters, resulting in a structure with exploitable porosity. Carefully designed MOFs are founded on the assembly of coordination linker units and directed bonding, where inherent defects are stacking faults or dislocations that form during the crystal growth process. Generally, the ligand and metal substitution process employing de novo synthesis and post-synthetic modification strategy can produce corresponding defects in MOFs. Moreover, the incorporation of additives during the synthesis of MOFs also leads to the breaking of connecting bonds in the metal centers without causing changes in the overall structure. Indeed, high ion mobility and low migration activation energy can be produced at vacant sites when the number of vacant sites exceeds the number of available ions [55, 161]. Consequently, the understanding of defect engineering also contributes to the further design and development of novel proton-conducting MOFs. From the structure of MOFs, the defective engineering is generally categorized into two types: (i) ligand defect and (ii) metal cluster defect.

#### Ligand Defect

**Fig. 12:**
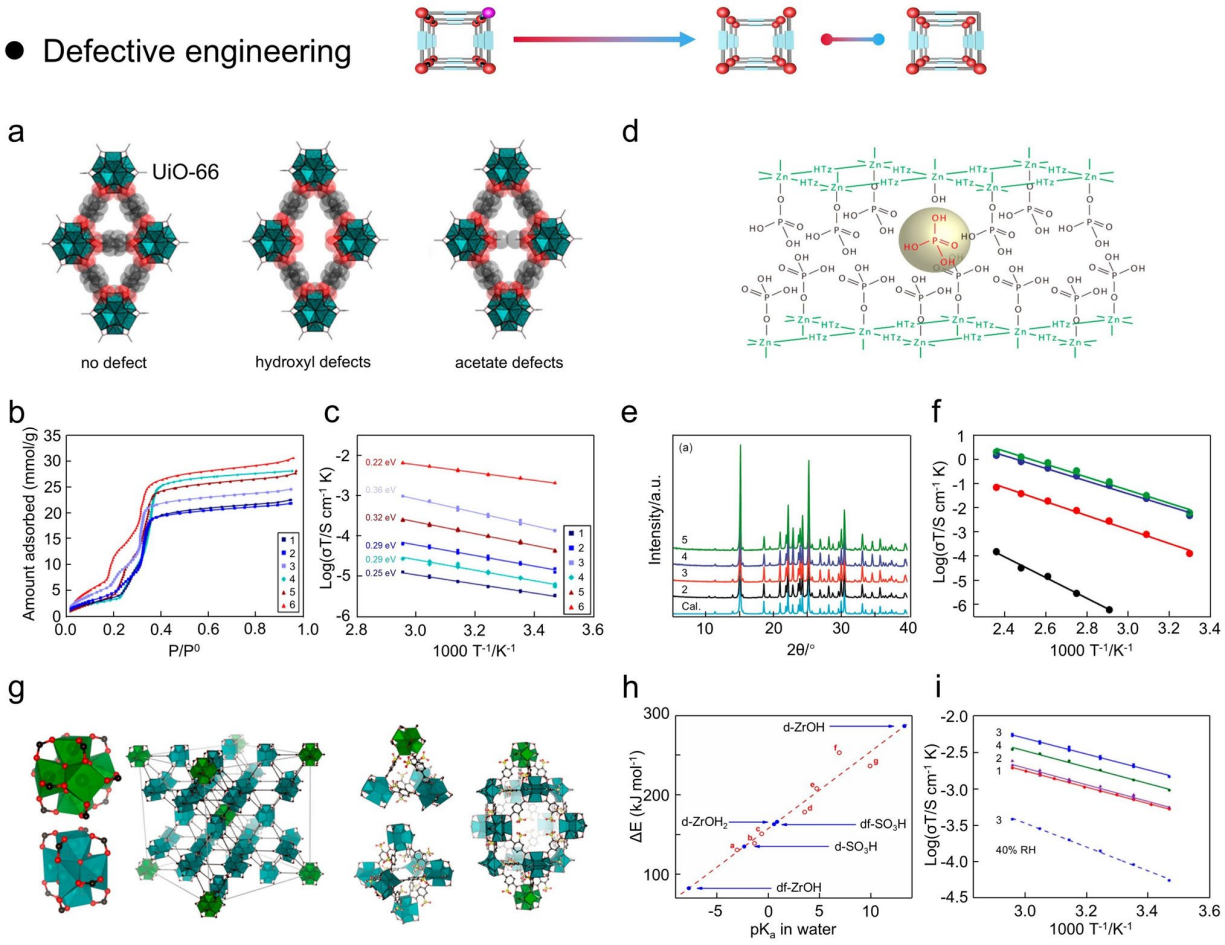
**a** Schematic diagram of ligand defects in UiO-66. **b, c** Water vapor adsorption and Arrhenius plot of samples 1–6. (Reproduced with permission from Ref.[163]. Copyright 2015, American Chemistry Society) **d** The structural model of 5. **e, f** XRD patterns and Arrhenius plot of 2, 3, 4, 5. (Reproduced with permission from Ref. [164]. Copyright 2016, American Chemistry Society) **g** Connectivity of zirconium clusters in sulfonated zirconium terephthalate within the unit cell, and differences between 12-connected and 9-connected clusters. **h** DFT calculation of d-ZrOH, d-ZrOH_2_, df-SO_3_H, d-SO_3_H, df-ZrOH for *pK*_*a*_ values in water. **i** Arrhenius plot of samples 1–4 at 95% RH (Reproduced with permission from Ref. [166]. Copyright 2015, American Chemistry Society)

Most approaches to enhance the proton conduction of MOFs have been through the use of ligands to increase their overall acidity (*e.g.*, phosphonic, sulfonic, carboxylic acid) or introduction of strong acids into the nanopores of MOFs [162]. Improving the pore acidity of MOFs can only resolve one factor that affects the proton conductivity–the charge carrier concentration. Indeed, the ion conductivity is the product of the ionic charge, carrier mobility, and carrier concentration. Ligand defects usually occur when an organic ligand is missing or the position is occupied by another small molecule. Specifically, the addition of several monocarboxylic acid molecules (such as HCOOH, CH_3_COOH, CH_3_CH_2_COOH, HCl, and C_2_HF_3_O_2_) can participate in coordination with metal ions during the synthesis of MOFs, leading to some molecules occupying the positions of the original ligands, and thus creating ligand defects. Generally, ligand defects in MOFs can be constructed by several strategies: (i) regulate the metal-to-ligand ratios; (ii) employ acid additives. From the perspective of proton mobility, Kitagawa and coworkers demonstrated that the incorporation of fatty acids to control the defective composition of the ligand during the synthesis of UiO-66 can significantly increase the concentration of charge carriers and proton mobility (Fig. 12a) [163]. UiO-66 consists of zirconium hexaoxide hydroxyl clusters that are linked by terephthalate linkers to create a cubic network of tetrahedral and octahedral micropores. Therefore, tuning the metal/ligand ratio or adding fatty acids as additives may create non-bridging ligand defects in the metal clusters, thus facilitating proton conduction. Notably, the amount of carboxylate defect per zirconium cluster measured by thermogravimetric analysis and elemental analysis were (1) 0.6, (2) 1.6, (3) 2.8, (4)1.4, (5) 0.4, (6) 1. The water absorption analysis proved that the samples 1–3 adsorbed 35.7, 34.0, and 36.2 mol of water per mole of formulation unit, while samples 4–6 adsorbed 45.7, 44.0, and 46.3 mol of water per unit, respectively, showing more water adsorption (Fig. 12b). Moreover, the proton conductivity increased with increasing concentration of sample defects to 1.3 × 10^–5^ S cm^−1^ in 1, 6.61 × 10^–5^ S cm^−1^ in 2, and 1.0 × 10^–3^ S cm^−1^ in 3 with maximum *E*_*a*_ values of 0.25, 0.29, and 0.36 eV at 65 °C, respectively, indicating that an increase from 5% ligand defects in 1 to 23% ligand defects in 3 results in a significant increase in proton conductivity of nearly 2 orders of magnitude. The proton conductivities of 4, 5, and 6 were 2.75 × 10^–5^, 2.63 × 10^–4^, and 6.93 × 10^–3^ S cm^−1^ (Fig. 12c). That is, defect engineering of MOF ligands can effectively improve proton conductivity.

Similarly, Inukai and coworkers demonstrated the realization of proton conductivity enhancement by incorporating proton carriers in a non-porous coordination polymer with defective sites [164]. In this 2D coordination polymer [Zn-(H_2_PO_4_)_2_(TzH)_2_]_n_, the metal centers are connected by TzH and the mono-dentate H_2_PO_4_^−^ ions are the main proton channel for proton conduction (Fig. 12d). As we mentioned above, as a general strategy to increase proton mobility and concentration, defects are induced at the H_2_PO_4_^−^ site and mobile H_3_PO_4_ species are encapsulated. In this process, differences in the amount of H_3_PO_4_ (4.0 for 2, 4.4 for 3, 4.8 for 4, and 5.2 mmol for 5) used to form defects lead to variations in the proportions of the elements in the framework. The absence of additional peaks of ZnO and HTz in XRD pattern suggests that the incorporation of H_3_PO_4_ cannot cause any change in the crystal structure of the coordination polymers (Fig. 12e). Moreover, the proton conductivity increased with the increasing percentage of H_3_PO_4_ as the temperature increased and the H_3_PO_4_ also encapsulation also further decreased the *E*_*a*_ value (0.85, 0.57, 0.52, and 0.53 eV for 2, 3, 4, 5, respectively) (Fig. 12f). For example, the proton conductivity of compound 5 at 150 °C is 4.6 × 10^–3^ S cm^−1^, which is four orders of magnitude higher than compound 2 and one order of magnitude higher than the defect-free compound.

Besides, Matoga and coworkers demonstrated the impact of introducing ligand defects in proton-conducting Zr-based MOFs (JUK-14) on the stability and proton conductivity [55]. JUK-14 is a layered Zr-based MOF where the Zr_6_O_4_(OH)_4_ cluster is linked by two sulfonic acid groups. Briefly, the JUK-14 variants with different numbers and types of defects (JUK-14_MeOH, JUK-14_H_2_O, JUK-14_HCl) were obtained by immersing the synthesized JUK-14 in methanol, distilled water, and 1 M HCl. Notably, the long-range ordered structure in the framework is essentially the same when transitioning from JUK-14 to JUK-4_MeOH and JUK-14_H_2_O, whereas the structure changes significantly when transitioning from JUK-14 to JUK-14_HCl (due to the complete removal of interlayer Me_2_NH_2_^+^). Notably, the proton conductivity of these three samples exhibits the following trends under the same conditions: JUK-4_MeOH > JUK-14_H_2_O < JUK-14_HCl, which suggests the replacement of Me_2_NH_2_^+^ in JUK-14 with proton is the critical factor in improving proton conductivity. Specifically, at 60 °C and 90% RH, the proton conductivities of JUK-4_ MeOH, JUK-14_HCl, and JUK-14_H_2_O were 1.8 × 10^–3^, 5.8 × 10^–4^, and 3.6 × 10^–4^ S cm^−1^, respectively. It is worth mentioning that the defective sites on the Zr clusters may saturate with terminal weakly acidic water molecules thus participating in proton conduction. On the other hand, JUK-14_MeOH also exhibits the lowest acidity. Therefore, it can be said the proton conductivity of MOFs is intricately and closely related to their acidity, water adsorption capacity, and thermal stability.

#### Metal Cluster Defect

The missing metal centers can offer hanging organic linkers and localized defects that become efficient proton conduction channels [165]. In general, the defects in metal centers are divided into intrinsic and extrinsic defects. The inherent defect is that the utilization of additives can partially replace the ligand without altering the overall structure during the synthesis of MOFs, allowing for fine-tuning of the properties [159]. The extrinsic defect is metal or ligand substitutions of the MOFs after synthesis. Briefly, the methods for preparing metal cluster defects are classified into the following two main types: (i) the addition of exotic metal precursors during the synthesis of MOFs competes with the target precursors for coordination; (ii) the replacement of some metal ions after the synthesis of target MOFs. In this regard, Kitagawa and coworkers reported sulfonated Zr-based MOFs with excellent proton conductivity and stability (Fig. 12g) [166]. The Zr-MOF with a hexanuclear Zr cluster was synthesized by 2-sulfoterephthalate and ZrCl_4_. Two types of metal clusters (9-connected clusters and 12-connected clusters) and three types of pores are present in the Zr-MOF. The proton conductivity is similar for the two metal cluster connection types with 1.93 × 10^–3^ and 1.82 × 10^–3^ S cm^−1^ at 65 °C and 95% RH, respectively. Notably, the proton conductivity of these samples is about one order of magnitude lower than that of other MOFs under the same measurement conditions, which may be due to the unsaturated coordination of metal clusters. Therefore, DFT calculations have been carried out using various models with defects (d) and without defects (df) to resolve the proton conduction phenomenon under low RH conditions. The results showed that *μ*_*2*_-oxide in the defect is a strong trapping site with a *pK*_*a*_ of 13.3, whereas the defect-free sites (df-ZrOH and df-SO_3_H) do not capture H^+^ (Fig. 12h and i).

### Guest Molecule Incorporation

To construct proton-conducing MOFs, the most common strategy is to encapsulate guest molecules (*e.g.*, H_2_SO_4,_ H_3_PO_4_, CsHSO_4_, CF_3_SOH, *p*-CH_3_C_6_H_4_SO_3_H, amino acids, imidazole, triazole) into nanopores. It is worth mentioning that achieving acidic molecule impregnation in coordination compounds is a challenge because the acid can attack the coordination framework. Therefore, the structural stability of host materials is a precondition for the successful impregnation of acidic guest molecules. Moreover, doping some non-acidic molecules (*e.g.*, triazole, imidazole, histamine, or metal–organic polyhedral) can also enhance the proton conductivities of MOFs effectively [152, 167–169].

#### Acid Guest Molecules

**Fig. 13:**
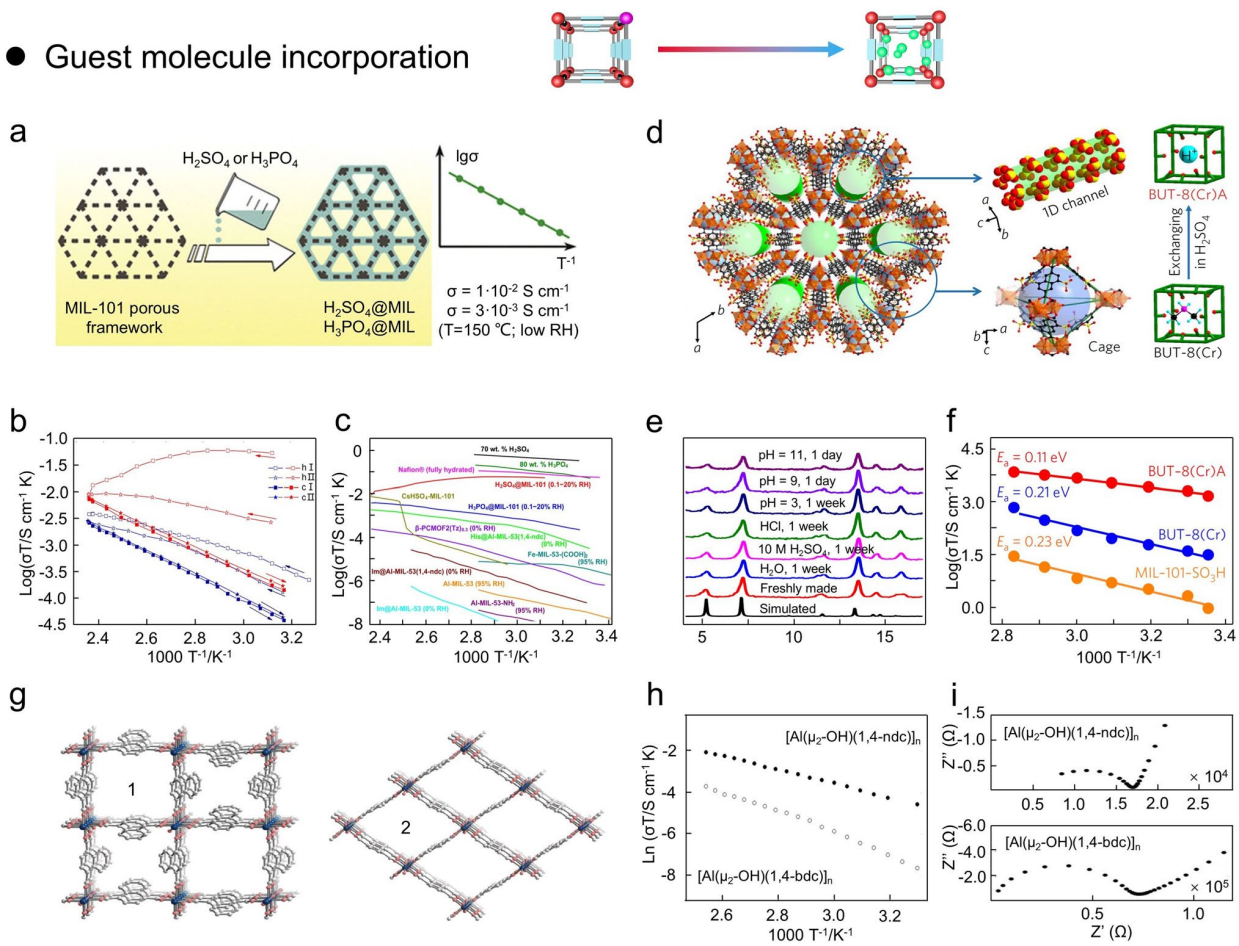
**a** Schematic diagram of doping H_2_SO_4_ or H_3_PO_4_ into MIL-101 to obtain high proton conductivity. **b, c** Arrhenius plots of H_3_PO_4_@MIL-101 (blue) and H_2_SO_4_@MIL-101 (red) as well as the summary of the proton conduction properties. (Reproduced with permission from Ref. [170]. Copyright 2012, American Chemistry Society) **d** Structure of BUT-8 and H_2_SO_4_-filled BUT-8(Cr). **e, f** XRD patterns of BUT-8(Cr) in acidic/basic conditions as well as the Arrhenius plots of BUT-8(Cr), BUT-8(Cr)A, and MIL-101-SO_3_H. (Reproduced with permission from Ref. [1]. Copyright 2009, Nature Publishing Group) **g** Structures of (1) and (2); **h, i** Arrhenius plots and Nyquist plots of 1-Im and 2-Im under anhydrous (Reproduced with permission from Ref. [129]. Copyright 2009, Nature Publishing Group)

Generally, the proton conduction of MOFs is dependent on the number and mobility of charge carriers (H^+^). Indeed, acid guest molecules enhance the proton conduction of MOFs in three main ways: (i) provide additional proton conduction sites. Acid guest molecules can provide multiple proton hopping sites within the pore of MOFs, allowing protons to move efficiently through the pore; (ii) formation of hydrogen bonding networks. Guest molecules can form a continuous network of hydrogen bonds with water molecules in the framework of MOFs, contributing to moving protons along a continuous path within the pore; (iii) regulation of proton conduction mechanisms. By introducing specific acid guest molecules, the proton conduction mechanism of MOFs may be changed from the traditional water-mediated proton conduction or transport mechanism to a more efficient hopping mechanism. In brief, acidic groups of acid guest molecules can induce more carrier aggregation and facilitate carrier transfer. Thus, the incorporation of acid guest into the structure of MOFs can significantly enhance the proton conductivity. In this regard, H_2_SO_4_ or H_3_PO_4_ with low volatility and strong acidity are the best candidates for acid guest molecules. Therefore, Fedin and coworkers demonstrated the realization of superior proton conduction using the impregnation of MIL-101 with H_2_SO_4_ and H_3_PO_4_ (Fig. 13a) [170]. Briefly, the H_2_SO_4_@MIL-101 (1) and H_3_PO_4_@MIL-101 (2) were synthesized by blending the MIL-101(Cr) with H_2_SO_4_ (2.7 M) or H_3_PO_4_ (2.6 M) and dried at elevated temperature. Considering the molar amount of acid, MIL-101 can confine about 70% H_2_SO_4_ in 1 and 80% H_3_PO_4_ in 2. As expected, the proton conductivities of 1 and 2 exhibited 4.0 × 10^–2^ and 2.5 × 10^–4^ S cm^−1^ at 25 °C and 20% RH. Considering the partially filled pores of MIL-101 and its non-conductive properties, these values match the conductivity of the corresponding liquid acid. It is worth noting that continuing to raise the temperature beyond 80 °C does not continue to improve the proton conductivity of 1 (6.0 × 10^–2^ S cm^−1^ at 80 °C). Moreover, at 150 °C, 1 and 2 exhibited the proton conductivities of 1.0 × 10^–2^ and 3.0 × 10^–3^ S cm^−1^, respectively (Fig. 13b). Notably, the proton conductivity of 1 is higher than 2, which demonstrates the positive effect of the acidity of the guest medium on the proton conductivity properties (higher *pK*_*a*_ values for H_2_SO_4_ than for H_3_PO_4_) (Fig. 13c). Similarly, Fedin and coworkers reported the addition of trifluoromethanesulfonic (TfOH) and toluenesulfonic (TsOH) into MIL-101 to obtain higher proton conductivity [171]. TfOH@MIL-101 and TsOH@MIL-101 were synthesized by adding MIL-101 into TfOH solution and TsOH solution, respectively. Notably, the proton conductivity of TfOH-containing solid is 8.0 × 10^–2^ S cm^−1^ at 60 °C and 15% RH, which is less than an order of magnitude away from that of H_3_PO_4_. Moreover, changes in the water content of TfOH@MIL-101 with humidity and temperature do not influence the proton conductivity much due to remaining water molecules and acid molecules that can remain in the proton conduction network. In contrast, the behavior of proton conductivity with humidity for TsOH@MIL-101 is quite different from TfOH@MIL-101. The proton conductivity of TsOH@MIL-101 was 1.8 × 10^–5^ S cm^−1^ at 25 °C, increasing to 3.0 × 10^–5^ S cm^−1^ at 50 °C, and decreasing to 8.0 × 10^–6^ S cm^−1^ at 66 °C.

In addition, Chen and coworkers presented a 3D Cr(III)-based flexible MOF (BUT-8 (Cr)A) with high proton conductivity (Fig. 13d) [1]. BUT-8(Cr) was synthesized through the reaction of Cr(NO_3_)_3_·9H_2_O and H_2_NDC(SO_3_H)_2_ under solvothermal conditions. The protonated BUT-8(Cr)A was obtained by washing and drying the BUT-8(Cr) by immersing it in an H_2_SO_4_ solution (0.5 M). Notably, the flexibility of BUT-8 (Cr)A allows it to cope with varying water contents in the pores and maintains efficient proton conduction even under low RH. Moreover, the flexible BUT-8(Cr)A can maintain superior integrity over a wide pH range and RH conditions due to the strong Cr–O bonds in Cr_3_O(CO_2_)_6_ (Fig. 13e). When comparing the conductivity with a rigid framework like MIL-101(Cr)-SO_3_H, BUT-8(Cr)A and BUT-8 (Cr) exhibited the proton conductivities of 6.3 × 10^–3^ and 1.3 × 10^–5^ S cm^−1^ at 65 °C, respectively. In particular, the proton conductivity of flexible framework MOFs and rigid framework MOFs at 11% RH shows a difference of two orders of magnitude. The *E*_*a*_ of MIL-101-SO_3_H, BUT-8(Cr), and BUT-8(Cr)A were 0.23, 0.21, and 0.11 eV at 100% RH, respectively (Fig. 13f).

Along the same research direction, Gao and coworkers selected UiO-66-COOH as host materials, glycine (Gly) and aspartic acid (Asp) as guest molecules to create a MOF-based proton conductor with excellent proton conductivity and cyclic stability [172]. UiO-66-COOH-Gly (1) and UiO-66-COOH-Asp (2) were prepared from UiO-66-COOH with Gly and Asp via amide reaction. At 30 °C and 98% RH, the proton conductivities of 1 and 2 were 4.24 × 10^–5^ and 2.62 × 10^–3^ S cm^−1^, respectively. Notably, the proton conductivity of 2 is an order of magnitude higher than that of 1 due to the greater abundance of carboxylic acid groups in 2. At 70 °C and 98% RH, the proton conductivities of UiO-66-COOH, 1, and 2 were 2.17 × 10^–4^, 4.90 × 10^–3^, and 1.19 × 10^–2^ S cm^−1^, respectively. In this regard, 2 exhibited the highest proton conductivity due to its highly efficient hydrogen bonding network constructed synergistically by water molecules, carboxylic acid groups, and amide groups. Briefly, amino acid-modified MOFs have superior proton conductivity and stability. However, the proton conductivity of amino acid-doped MOFs is lower than that of inorganic strong acid-doped MOFs under the same conditions due to the lower acidity of amino acid-based molecules.

#### Other Molecules

As we mentioned above, the addition of imidazole, triazole, histamine, metal–organic polyhedral, and polyoxometalate to the pores of MOFs also allows the construction of proton-conducting MOFs, especially at high temperatures. In this regard, Kitagawa and coworkers achieved exceptional anhydrous proton conductivity by filling 1D channels of two aluminum-based coordination polymers with imidazole molecules [129]. The low conductivity of solid imidazole is due to the strong hydrogen bonding interactions that reduce the mobility of the molecule thereby adversely affecting the proton conduction. That is, providing an ideal transport space for carrier molecules and increasing carrier mobility is essential to facilitate proton conduction (Fig. 13g). Considering the shape and size of imidazole, the aluminum compounds [Al(*μ*_2_-OH)(1,4-ndc)]_n_ (1) and [Al(*μ*_2_-OH)(1,4-bdc)]_n_ (2) were chosen to encapsulate the imidazole molecules. Specifically, the structures of 1 and 2 consist of numerous AlO_4_(*μ*_2_-OH)_2_ chains that are interconnected by dicarboxylic acid ligands to construct a 3D framework with 1D nanochannels. The thermogravimetric curves showed imidazole loadings of 14% weight and 30% weight for 1 and 2, respectively. Moreover, the proton conductivity of 1-Im can reach 5.5 × 10^–5^ S cm^−1^ at 25 °C, which is in the same order of magnitude as that of solid intrinsic imidazole. Meanwhile, the proton conductivity of 1-In was 2.2 × 10^–5^ S cm^−1^ at 120 °C (*E*_*a*_ = 0.6 eV). Under the same conditions, the 2-In had a proton conductivity of 10^–10^ S cm^−1^ at 25 °C and 1.0 × 10^–7^ S cm^−1^ at 120 °C (*E*_*a*_ = 0.9 eV) (Fig. 13h, i). Notably, the amount of imidazole loaded by 2-Im is higher than 1-Im, but the proton conductivity of 2-Im is lower than that of 1-Im by about two orders of magnitude. Briefly, the microchannels within compound 1 exhibit nonpolar potential surface, allowing the polar imidazole to move unrestrictedly within this channel as it does not strongly interact with the host framework. In contrast, imidazole with strong interactions with 2 cannot rotate or move easily in the pores due to the dense stacking and strong host–guest interactions.

### Pore-Space Manipulation

The configuration and environment of pores are intricately linked to the choice of functional groups in ligands. As we mentioned above, the simplest pore treatment to prepare proton-conducting MOFs is to incorporate the conducting medium into their void space. In general, the representative proton-conducting MOFs are associated with the addition of acidic guests or amphoteric guest molecules. In this regard, both hydrophilic/hydrophobic surfaces of MOFs can influence the hydrogen bonding interactions between the conducting medium and the framework surface, thus improving the proton conductivity and conduction mechanism. Indeed, the hydrophilicity of MOFs affects the hydrogen bonding interactions between the conducting medium and the framework, thus affecting the proton conduction properties [173]. In brief, water-mediated proton conductors typically require high humidity conditions for efficient operation, while anhydrous proton conductors require high temperatures to produce mobile proton species with high conductivity. Generally, proton-conducting MOFs have a similar tendency and do not have high proton conductivity under low temperature and humidity conditions. That is, the conductivities of MOFs are proportional to temperature and humidity. However, hydrophilicity is a key factor in water affinity, and excellent hydrophilicity enables MOFs to induce efficient proton conduction even at relatively low humidity. Therefore, in this section, we focus on the effect of hydrophilicity and hydrophobicity of MOFs on proton conduction and try to resolve the connection between the pore space of MOFs and proton conduction from a new perspective.

#### Hydrophilic

**Fig. 14:**
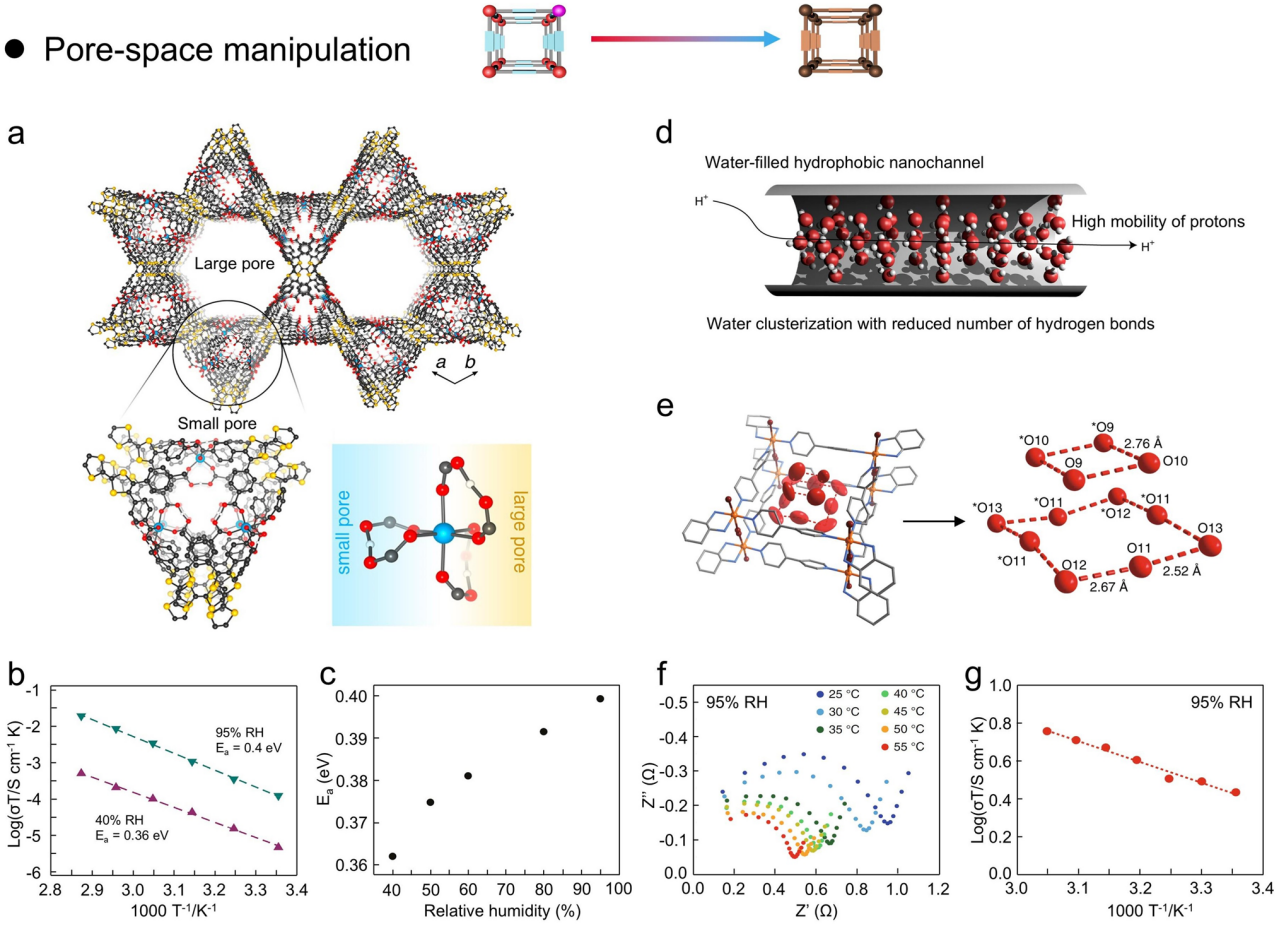
**a** Small and large pore structure of MIT-25. **b, c** The Arrhenius plots of MIT-25 at 40% RH and 95% RH and activation energy as a function of RH. (Reproduced with permission from Ref. [174]. Copyright 2018, American Chemistry Society) **d** Schematic diagram of proton transport through a hydrophobic nanochannel filled with water. **e** Tetramer and octamer water clusters of [Pt(dach)(bpy)Br]_4_(SO_4_)_4_·32H_2_O. **f, g** Nyquist plots and Arrhenius plots of [Pt(dach)(bpy)Br]_4_(SO_4_)_4_·32H_2_O under 95% RH (Reproduced with permission from Ref. [120]. Copyright 2020, Nature Publishing Group)

Hydrophilicity, a critical factor in water affinity, allows MOFs to produce efficient proton conduction at low RH conditions. Controlling the hydrophilicity of pore surfaces is of comparative significance for the design of proton-conducting MOFs that operate under mild conditions. In this regard, Dinca and coworkers investigated the proton conduction properties of MOFs (MIT-25) with two different hydrophilic nanopores (Fig. 14a) [174]. Mesoporous MIT-25 with two different hydrophilic channels (the diameters of large and small pores were 27 and 4.5 Å) was synthesized via Mg^2+^ and tetrathiafulvalene-tetrabenzoate (TTFTB^4−^) ligand. The large pores are surrounded by *μ*_2_-H-bridged carboxylic acids coordinated to Mg^2+^, and the small pores include additional *μ*_2_-H-bridged carboxylic acids and H_3_O^+^ oriented toward the pores, both of which exhibit characteristic step adsorption behavior in water vapor adsorption. In brief, the first process occurs in hydrophilic small pores below 40% RH, while the second process occurs in hydrophilic large pores above 50% RH. The proton conductivity of MIT-25 was investigated using a pelletized sample under various humidity and temperature conditions. For example, MIT-25 exhibited the proton conductivities of 1.58 × 10^–5^ and 1.03 × 10^–4^ S cm^−1^ at 25 and 75 °C under 40% RH (*E*_*a*_ = 0.36 eV), respectively (Fig. 14b, c). That is, the small pores are occupiable by guest water molecules at a relative humidity of 40%, suggesting that the proton conduction behavior at low RH is primarily attributable to the Grotthuss mechanism in hydrophilic pores. Nevertheless, the proton conductivity of MIT-25 at high RH conditions involves averaging large and small pores, which is the result of water molecules filling the large and small pores sequentially. Notably, despite the difficulty of definitively explaining the proton conduction mechanism in MIT-25 large nanopores in terms of *E*_*a*_ values, the increase in *E*_*a*_ values with increasing RH explains the predominance of carrier diffusion. Briefly, the high hydrophilicity of MOFs will induce more water molecules to aggregate around the framework, which enhances the proton conduction by increasing the carrier concentration near the framework. In other words, the humidity correlation between hydrophilicity and proton conductivity highlights the importance of pore size properties.

#### Hydrophobic

Notably, the water molecules confined in 1D hydrophobic nanochannels are of interest because of their unusual structural and dynamic properties. Briefly, the confined water molecules show remarkable physical properties in hydrophobic nanochannels with smaller pore sizes because of the reduced number of adjacent water molecules forming a confined network of hydrogen bonds [175, 176]. To resolve the conductive behavior of water molecules in hydrophobic nanochannels, Kitagawa and coworkers reported a distinctive water cluster structure and high proton conductivity directly observed in hydrophobic metal–organic nanotubes (MONs), [Pt(dach)(bpy)Br]_4_(SO_4_)_4_·32H_2_O (Fig. 14d) [120]. The MONs were fabricated by the oxidation polymerization of Br_2_ with Pt. Briefly, the Pt is attached to the bpy ligand to form the rectangle [Pt(dach)(bpy)]_4_ with a net charge of + 8, in which the axial sites of Pt are linked by Br atoms to form the entire MONs. Notably, there are two types of water-filled 1D nanochannels in the crystal structure of MONs, an internal hydrophobic channel and an external hydrophilic channel. In the hydrophobic channel, water molecules were held close to each other with an O–O spacing of 2.5–2.7 Å and weak hydrogen bonds of 3.0–3.4 Å between clusters. The distance between the water molecules inside the channel and the channel wall is more than 2.9 Å, which means that the interaction is weak. In the hydrophilic channel, water molecules are formed into a 1D hydrogen bonding network that involves sulfate anion and amino group (Fig. 14e). Notably, the proton conductivity of MONs was 1.7 × 10^–2^ S cm^−1^ at 55 °C and 95% RH, which is two orders of magnitude higher than that of pelletized samples (Fig. 14f, g). Moreover, ^1^H-NMR measurements were used to obtain a deeper understanding of the proton conduction mechanism since the self-diffusion coefficient of 1H in water is linearly related to the mobility of proton carriers during proton conduction. The simulated calculated proton diffusivities showed that the vehicle diffusion coefficient and Grotthuss diffusion coefficient in hydrophobic channels were 1.5 and 2.0 times higher than in hydrophilic channels, respectively. That is, high proton conductivity without the introduction of strongly acidic molecules in hydrophobic channels.

The structural features, testing conditions, performance comparisons, and construction strategies of the reported proton-conducting MOFs are summarized in Table 1. Further, the properties of MOF-based proton exchange membranes for fuel cells and flow batteries are summarized in Table 2.

**Table 1:** Comparison of proton-conducting MOFs

Compound	Structure	Conditions	Conductivity (S cm^−1^) (*E*_*a*_)	Strategy	References
PCMOF-20(DMA)_3_[Zr(HL)F_2_]	2D	80 °C and 95% RH	1.0 × 10^–2^ (0.2 eV)	Counterion	[177]
ZH[Mg(H_2_O)_6_][NaFe_x_Al_1−x_(C_2_O_4_)_3_]	2D	25 °C and 90% RH	3.0 × 10^–3^ (0.37 eV)	Counterion	[74]
NMe_3_(CH_2_COOH)[FeCr(ox)_3_]	2D	25 °C and 65% RH	8.0 × 10^–4^	Counterion	[56]
[NH(prol)_3_][MCr(ox)_3_]	2D	25 °C and 75% RH	1.0 × 10^–4^ (0.37 eV)	Counterion	[178]
Ti-CAT-5	3D	25 °C and 98% RH	8.2 × 10^–4^ (0.43 eV)	Counterion	[179]
VNU-15	3D	95 °C and 60% RH	2.9 × 10^–2^ (0.22 eV)	Counterion	[133]
POMOF	3D	80 °C and 75% RH	1.04 × 10^–2^ (0.22 eV)	Counterion	[180]
[(Me_2_NH_2_)_3_(SO_4_)_2_][Zn_2_(ox)_3_]	3D	25 °C and 98% RH	4.2 × 10^–2^	Counterion	[80]
MROF-1	3D	70 °C and 97% RH	1.72 × 10^–2^ (0.37 eV)	Counterion	[181]
(N_2_H_5_)[CeEu(C_2_O_4_)_4_(N_2_H_5_)]·4H_2_O	3D	25 °C and 100% RH	3.42 × 10^–3^ (0.10 eV)	Counterion	[182]
Li-HPAA	1D	24 °C and 98% RH	1.10 × 10^–4^ (0.84 eV)	Ligand functionalization	[183]
[Zn(H_2_PO_4_)_2_(HPO_4_)]·H_2_dabco	1D	160 °C	8.0 × 10^–8^ (1.2 eV)	Ligand functionalization	[184]
[Zn_3_(H_2_PO_4_)_6_]·(Hbim)	1D	120 °C	1.3 × 10^–3^	Ligand functionalization	[185]
MIL-53(Al)-NH_2_	3D	80 °C and 95% RH	4.1 × 10^–8^ (0.45 eV)	Ligand functionalization	[139]
[Zn(HPO_4_)(H_2_PO_4_)_2_]·(ImH_2_)_2_	1D	130 °C	2.6 × 10^–4^ (0.47 eV)	Acid group	[68]
PCMOF3	2D	25 °C and 95% RH	3.5 × 10^–5^ (0.17 eV)	Acid group	[186]
MFM-500	2D	25 °C and 98% RH	4.5 × 10^–4^ (0.17 eV)	Acid group	[187]
Na-HPAA	3D	24 °C and 98% RH	5.6 × 10^–3^ (0.39 eV)	Acid group	[183]
Ca-PiPhtA-I	3D	24 °C and 98% RH	5.7 × 10^–4^ (0.32 eV)	Acid group	[188]
JUC-200	3D	80 °C and 98% RH	1.62 × 10^–3^ (0.23 eV)	Acid group	[189]
SrSBBA	2D	25 °C and 98% RH	4.4 × 10^–5^ (0.56 eV)	Acid group	[190]
BUT-8(Cr)A	3D	80 °C and 100% RH	1.27 × 10^–1^ (0.11 eV)	Acid group	[1]
PCMOF2	3D	80 °C and 100% RH	1.17 × 10^–1^ (0.22 eV)	Acid group	[87]
TMOF-2	3D	90 °C and 98% RH	1.23 × 10^–4^ (0.37 eV)	Acid group	[191]
MIP-177-SO_4_H-LT	3D	25 °C and 95% RH	2.6 × 10^–2^	Acid group	[192]
BUT-83	3D	80 °C and 97% RH	3.9 × 10^–2^ (0.34 eV)	Acid group	[193]
Urea-MOF-74	3D	55 °C and 95% RH	3.69 × 10^–2^ (0.14 eV)	Metal center	[155]
Cr-MIL-88B-PESE	3D	100 °C and 85% RH	4.50 × 10^–2^ (0.34 eV)	Metal center	[156]
Im-Fe-MOF	3D	60 °C and 98% RH	4.50 × 10^–2^ (0.44 eV)	Metal center	[153]
Mg-OBA	2D	80 °C and 95% RH	1.27 × 10^–2^ (0.13 eV)	Metal center	[194]
([Co_2_Cl_2_(BTC)_4/3_(Me_2_NH_2_)+_2_·4/3H_2_O])_n_	3D	50 °C and 60% RH	1.19 × 10^–3^ (0.27 eV)	Guest inclusion	[195]
H_3_PO_4_@MIL-101	3D	150 °C	3.0 × 10^–3^ (0.25 eV)	Guest inclusion	[170]
H_2_SO_4_@MIL-101(Cr)	3D	150 °C	1.0 × 10^–2^ (0.42 eV)	Guest inclusion	
H^+^@Ni_2_(dobdc)[Ni_2_-(dobdc)(H_2_O)_2_]·6H_2_O	3D	80 °C and 95% RH	2.2 × 10^–2^ (0.25 eV)	Guest inclusion	[196]
His@NVU-23	3D	95 °C and 85% RH	1.79 × 10^–2^ (0.27 eV)	Guest inclusion	[197]
Im@UiO-67	3D	120 °C	1.44 × 10^–3^ (0.36 eV)	Guest inclusion	[198]
Im@NENU-3	3D	70 °C and 90% RH	1.82 × 10^–2^ (0.57 eV)	Guest inclusion	[154]
Im@MOF-808	3D	65 °C and 99% RH	3.4 × 10^–2^ (0.25 eV)	Guest inclusion	[152]
Im@Fe-MOF	3D	60 °C and 98% RH	4.23 × 10^–3^ (0.57 eV)	Guest inclusion	[153]
Im@[Al(*μ*_2_-OH)(1,4-ndc)]	3D	120 °C	2.2 × 10^–5^ (0.60 eV)	Guest inclusion	[129]
Tz@*β*-PCMOF2	3D	150 °C	2.0 × 10^–4^ (0.51 eV)	Guest inclusion	[123]
CDMOF-2	3D	25 °C and MeOH vapor	4.8 × 10^–6^	Guest MeOH	[199]
NH4Br@HKUST-1	3D	25 °C and 99% RH	8.99 × 10^–4^ (1.42 eV)	Guest inclusion	[200]
Defect [Zn(H_2_PO_4_)2HTz_2_]_n_	2D	30 °C and 98% RH	2.0 × 10^–2^ (0.53 eV)	Defect	[166]
Mg_2_H_6_(H_3_O)(TTFTB)_3_	3D	75 °C and 95% RH	5.1 × 10^–4^ (0.40 eV)	Hydrophilic pore	[174]
NMe_3_(CH_2_COOH)[FeCr(ox)_3_]	2D	25 °C and 65% RH	8.0 × 10^–4^	Hydrophilic pore	[56]
[Pt(dach)(bpy)Br]_4_(SO_4_)_4_·32H_2_O	1D	55 °C and 95% RH	1.7 × 10^–2^ (0.22 eV)	Hydrophobic pore	[120]

**Table 2:** Summary of MOF-based proton exchange membranes for the performance in energy-related applications

Applications	Membranes	Conductivity (S cm^−1^)	Tensile strength (MPa)	Maximum power density (mW cm^−2^)	References
Hydrogen–oxygen fuel cells	PEM-1	1.1 × 10^–2^ (50 °C and 99% RH)	–	853 (50 °C and 15% RH)	[201]
	N_U200-2	2.7 × 10^–1^ (110 °C and 95% RH)	11.9	–	[202]
	SPEEK/HPW@MIL-101	6.5 × 10^–3^ (60 °C and 40% RH)	–	235 (60 °C and 55% RH)	[203]
	MOF-801@PP	1.84 × 10^–3^ (52 °C and 98% RH)	-	2.2 (30 °C and 100% RH)	[204]
	IM-UiO-66-AS@PP	1.19 × 10^–2^ (80 °C and 98% RH)	4.62	17.5 (80 °C and 98% RH)	[205]
	IL@MOF-808 membrane	2.24 × 10^–2^ (70 °C and 98% RH)	5.57	–	[206]
	SO_3_H-IL-PMo_12_@MIL-101 membrane	7.3 × 10^–3^ (70 °C and 98% RH)	2.23	0.93 (30 °C and 98% RH)	[207]
	CS/H_2_SO_4_@MIL-101–8	9.5 × 10^–2^ (100 °C and 100% RH)	55.5	146 (80 °C)	[208]
	CS/UiO-66-SO_3_H + UiO-66-NH_2_	3.78 × 10^–3^ (120 °C)	–	10.6 (120 °C)	[209]
	PFSA/Ce-BTC	1.95 × 10^–1^ (80 °C and 100% RH)	–	1710 (75 °C and 80% RH)	[210]
	CP/MOF membrane	4.4 × 10^–3^ (90 °C and 100% RH)	–	25 (80 °C and 100% RH)	[211]
Direct methanol fuel cells	GO@UiO-66-NH_2_/Nafion	3.03 × 10^–1^ (90 °C and 95% RH)	–	–	[212]
	UiO-66-NH_2_@NFs-8/Nafion	2.7 × 10^–1^ (80 °C and 100% RH)		95.49 (60 °C and 100% RH)	[213]
	F-UN-5.0%@Nafion	2.5 × 10^–1^ (80 °C and 100% RH)	35.8	26.8	[214]
	IL-MOF-1 membrane	1.84 × 10^–1^ (80 °C and 100% RH)	–	37.5 (80 °C and 2 M methanol)	[215]
	MNCS@SNF-PAEK-1.5	1.88 × 10^–1^ (80 °C and 100% RH)	36.24	90.8 (80 °C and 100% RH)	[216]
	DNA@ZIF-8 membrane	1.7 × 10^–1^ (75 °C and 97% RH)	–	9.87 (80 °C and 1 M methanol)	[217]
Flow batteries	S-808–3	7.66 × 10^–2^ (75 °C and 97% RH)	–	93.7% voltage efficiency at 40 mA cm^−2^	[218]
	IM-UIO-66-AS/SPEEK	12.2 × 10^–3^	–	79.9% voltage efficiency at 200 mA cm^−2^	[219]
	S/PDA@808-10 min	–	40	85.3% voltage efficiency at 120 mA cm^−2^	[220]
	CuBTC/Celgard	–	–	60.7 mW cm^−2^ at 36 mA cm^−2^	[221]
	Nf/S-U66-5	6.42 × 10^–2^	3.46	91.5% voltage efficiency at 80 mA cm^−2^	[222]

## Conclusion and Perspectives

**Fig. 15:**
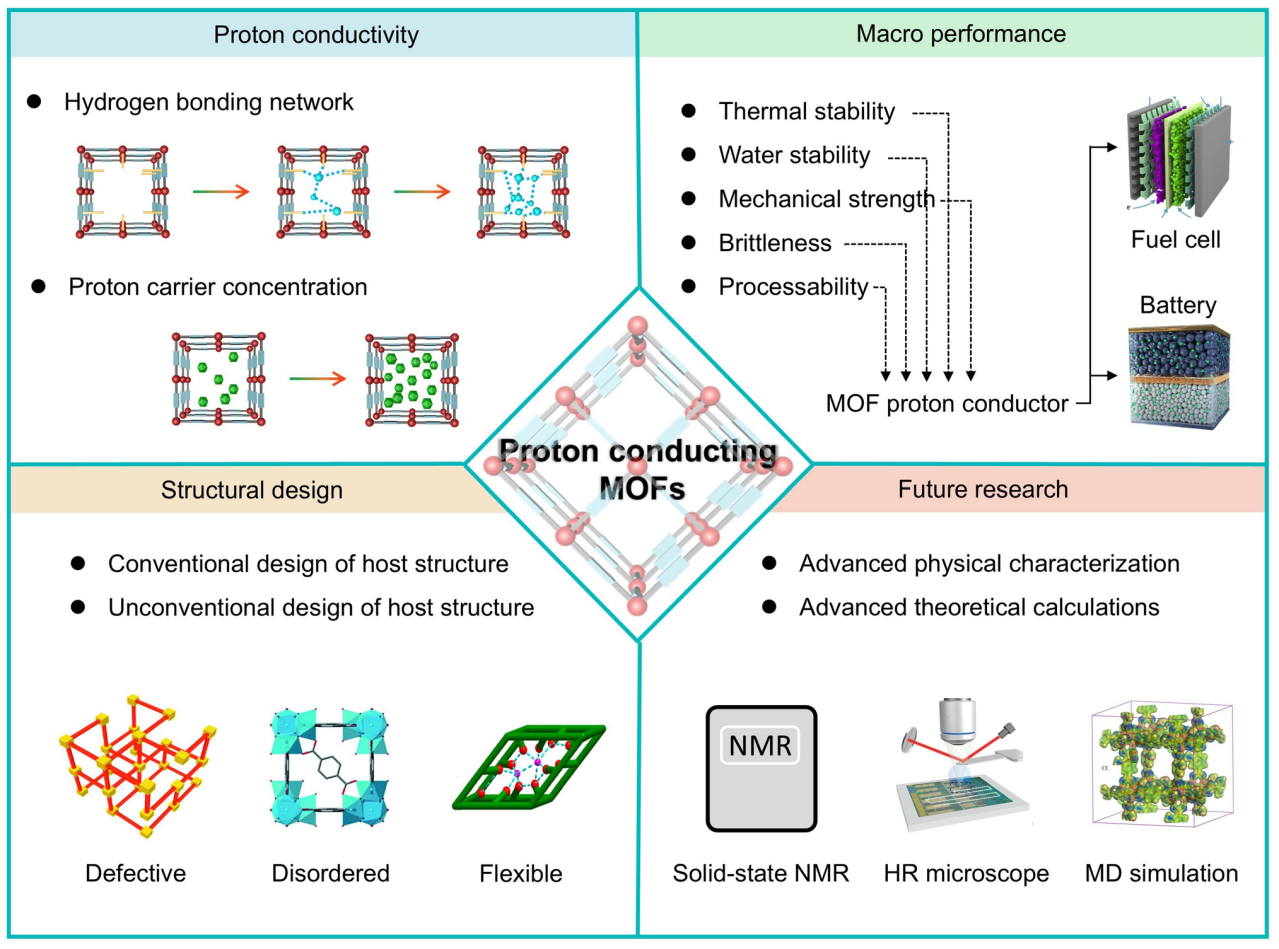
Key features and developmental perspectives of proton-conducting MOFs

MOFs with well-defined crystal structures, interesting structural tunability, and tailorable porosity have shown tremendous potential and particular advantages for proton conduction applications, and thus have received considerable attention. In this review, we comprehensively summarize the impact of the dimensional structures and properties of MOFs on the proton conduction behavior, and present a detailed discussion of the strategies for constructing proton-conducting MOFs through representation examples. From the previous results, the key features for constructing proton-conducting MOFs are as follows (Fig. 15):

(i) Constructing a continuous hydrogen bonding network. Typically, the proton conduction of MOFs is strongly affected by humidity conditions. Especially at low humidity conditions, the continuous proton conduction pathway formed through hydrogen bonding is readily disrupted, resulting in low proton conductivity. In this regard, two approaches have been employed to address this problem: Designing intrinsic hydrogen bonding networks and developing novel alternative conduction media. Indeed, MOFs can be classified into three categories according to structure and pore characteristics: (a) The unstable MOFs have no permanent pores removal of guest molecules; (b) the rigid MOFs can keep a robust porous framework under various conditions; (c) the flexible MOFs allow for a reversible response to external stimuli while maintaining the integrity of the framework. That is, a flexible structure that can be structurally transformed in response to changes in humidity may exhibit better resistance to humidity-dependent proton conductivity.

(ii) Increasing the concentration of proton carriers (matched *pK*_*a*_ values in the proton conduction pathways). Generally, the proton source of the carrier concentration comes from the guest molecules (*e.g.*, water molecules, organic molecules, and acid molecules), counter-cations (*e.g.*, NH_4_^+^, H_3_O^+^, and Me_2_NH_2_^+^), and functional groups of MOFs. For example, H_2_SO_4_ has a low *pK*_*a*_ value and is the best candidate for achieving high proton conductivity and low *E*_*a*_. Notably, the introduction of low *pK*_*a*_ species into the nanopores of MOFs can indeed be effective in enhancing the proton conduction, but the long-term cyclic stability of the conductivity in this case may receive a certain degree of constraint. In this regard, the structural integrity and morphological stability of the MOFs are factors that need to be considered emphatically. That is, the design and development of MOF host materials with enhanced stability is a top priority.

Additionally, although significant progress has been achieved in proton-conducting MOFs, there remains considerable work to be done before they can be effectively utilized in proton exchange membrane fuel cells. Specifically, several critical factors need to be fully considered when assembling MOF proton conductors into fuel cells, including thermal and water stability, mechanical strength and brittleness, and processability. From this perspective, the proton-conducting MOFs are required to fulfill the following demands: (a) The proton conduction in MOFs requires breaking the limits of humidity and temperature to match the industrial applications. For example, fuel cells in automotive applications require the PEMs to operate at temperatures of approximately 120 °C, whereas in stationary applications, limited water distribution requires PEMs to operate at temperatures higher than 120 °C. Notably, the proton conductivity will decrease dramatically without the proper construction of water channels to promote the generation of H_3_O^+^ that hops between H_2_O molecules under low humidity conditions. Indeed, high water content or humidity facilitates rapid proton conduction, whereas this property is limited by the poor stability of MOFs. To reduce the effect of MOF stability on proton conductivity, water-stability, and ultra-stabilized MOFs were constructed by enhancing the ligand rigidity, reducing the contact between the MOF body and water molecules, and increasing the strength of ligand bonds; (b) the brittleness of MOFs can be effectively strengthened by choosing metal clusters or organic ligands that can create more robust coordination interactions, or by filling the nanopores with proton-conducting guests; (c) the application of MOFs as membrane separators in fuel cells requires focused attention. In fact, solid MOFs are usually not directly used as separators due to their fragile properties. In this regard, alkyl chains and linear polymer guests on organic ligands may facilitate to enhance the processability of MOFs. Moreover, the design of novel MOF membranes with high stability may have great promise in this regard.

For proton-conducting MOFs, future research may need to be focused on the following areas: (a) The elaborate design of host structure remains the priority for the construction of novel proton-conducting MOFs. The spatial structure, stability, and proton conductivity of MOFs can be altered accordingly by the embedment of different organometallic groups. Moreover, more advanced strategies such as constructing defective or disordered MOFs, designing interpenetrating MOFs, and introducing novel proton carriers should be explored. (b) Some MOFs with unique structures (*e.g.*, disordered MOFs, defective MOFs, or flexible MOFs) may have higher proton conduction properties than conventional MOFs with regular and rigid structures. In general, synthesizing MOFs with unique structures require various special ligands or different post-synthetic treatments. It is worth noting that these particular ligands and treatments lead to some degree of unpredictability in the construction of framework structures. (c) Proton-conducting MOFs are generally performed by EIS measurements of proton conductivity to further extrapolate the possible proton conduction mechanism. It needs to be noted that the detailed pathways for proton conduction are different in each system, and extrapolation of the corresponding conduction mechanism from *E*_*a*_ along may be deviant. Moreover, these deviations are difficult to verify because of the absence of theoretical calculations and precise characterization measurements, which leads to a lag in the research of actual ion mobility. In this regard, some advanced physical-dynamics characterization methods (*e.g.*, molecular dynamic computations, solid-state nuclear magnetic resonance, and neutron scattering) can be employed to accurately characterize the proton conduction mechanism.

Overall, the proton-conducting MOFs have made tremendous progress over the past decade. In a narrow sense, it is fascinating to analyze the proton conduction process in MOFs, and in a broader sense, an enhanced understanding of proton-conducting MOFs is essential to facilitate their application in energy storage and conversion technologies. It is foreseeable that with the intensive cooperation of researchers in various fields, more significant breakthroughs in novel proton-conducting MOFs are expected to be achieved shortly.
